# Pitfalls in quantitative myocardial PET perfusion II: Arterial input function

**DOI:** 10.1007/s12350-020-02074-8

**Published:** 2020-03-03

**Authors:** Linh Bui, Danai Kitkungvan, Amanda E. Roby, Tung T. Nguyen, K. Lance Gould

**Affiliations:** 1 Department of Medicine, Division of Cardiology and Weatherhead PET Center For Preventing Atherosclerosis, McGovern Medical School and Memorial Hermann Hospital, Houston, TX USA; 2 Weatherhead PET Center for Preventing Atherosclerosis, McGovern Medical School and Memorial Hermann Hospital, Houston, TX USA; 3 Programming and Data Management, Weatherhead PET Center, McGovern Medical School, University of Texas, Houston, TX USA; 4 Weatherhead PET Center For Preventing and Reversing Atherosclerosis, McGovern Medical School, University of Texas Health Science Center at Houston, 6431 Fannin St., Room MSB 4.256, Houston, TX 77030 USA

**Keywords:** Quantitative myocardial perfusion, cardiac PET

## Abstract

**Rationale:**

We aimed to define the impact of variable arterial input function on myocardial perfusion severity that may misguide interventional decisions and relates to limited capacity of 3D PET for high-count arterial input function of standard bolus R-82.

**Methods:**

We used GE Discovery-ST 16 slice PET-CT, serial 2D and 3D acquisitions of variable Rb-82 dose in a dynamic circulating arterial function model, static resolution and uniformity phantoms, and in patients with dipyridamole stress to quantify per-pixel rest and stress cc·min^−1^·g^−1^, CFR and CFC with (+) and (−) 10% simulated change in arterial input.

**Results:**

For intermediate, border zone severity of stress perfusion, CFR and CFC comprising 7% of 3987 cases, simulated arterial input variability of ± 10% may cause over or underestimation of perfusion severity altering interventional decisions. In phantom tests, current 3D PET has capacity for quantifying high activity of arterial input and high-count per-pixel values of perfusion metrics per artery or branches.

**Conclusions:**

Accurate, reproducible arterial input function is essential for at least 7% of patients at thresholds of perfusion severity for optimally guiding interventions and providing high-activity regional per-pixel perfusion metrics by 3D PET for displaying complex quantitative perfusion readily understood (“owned”) by interventionalists to guide procedures.

**Electronic supplementary material:**

The online version of this article (10.1007/s12350-020-02074-8) contains supplementary material, which is available to authorized users.

## Introduction

Positron emission tomography (PET) quantifies regional absolute myocardial perfusion in cc·min^−1^·g^−1^, coronary flow reserve (CFR) and their combination as coronary flow capacity (CFC) associated with reduced mortality after revascularization compared to medical treatment^1^ and differentiates focal, diffuse, and small vessel disease.^2^ The arterial input function is widely recognized as essential for quantitative myocardial perfusion^3^ with an extensive literature that, however, does not resolve several issues for Rb-82 imaging.

Currently, arterial input function for myocardial perfusion models by PET is measured by two perfusion models. The most traditional, widely used model generates time activity curves in regions of interest (ROI) on serial, short-duration, and first pass images. The ROI is located by back projection from later myocardial images onto first pass images targeting the left atrium or the LV atrio-ventricular valve plane. These time activity curves are fit to a compartmental perfusion model to solve for perfusion values that best fit observed arterial and myocardial time activity curves.^4^,^5^

Alternatively, a validated “simple” or “retention” perfusion model uses a fixed arterial phase image (2 minutes for Rb-82) followed by fixed myocardial phase acquisition (5 minutes for Rb-82) 5-12 accounting for flow dependent extraction of Rb-82. It is validated experimentally^5^ as equivalent to compartmental modeling for quantitative perfusion and has substantial clinical application.^5^-^12^

Although not widely used, from one viewpoint, the “simple” or “retention” model has two advantages. First, it provides high-quality, high-count arterial phase images for precise location of an arterial phase ROI directly on the maximal activity in left atrium or aortic root throughout the dynamically moving and translating heart commonly of 2 cm or more during vasodilator stress, systole and diastole.^7^ This heart translation may move the heart in and out of an estimated, fixed, and back projected ROI from myocardial images thereby causing substantial variability of arterial input and corresponding variability in quantitative perfusion.^7^ While multicompartmental models are traditional and most widely used, direct objective comparison of the two models suggests that the retention model is a valid alternative since “the retention model may have higher sensitivity for detection and localization of abnormal flow and MFR using Rb-82 and N-13 ammonia.” .^4^

Secondly, the simple or retention model (i) is highly reproducible ±10% on test/retest measurement in the same patient within minutes,^6^ (ii) has a well-documented threshold severity for stress induced angina or ST depression^9^,^11^, and (iii) predicts high risk of death or myocardial infarction^1^,^2^ that is significantly reduced by revascularization.^1^ However, sensitivity analysis for effects of variable arterial input on severity for guiding interventions needs objective definition.

Despite accounting for most literature on quantitative myocardial perfusion, established 2D/3D PET-CT scanners using BGO (bismuth germanate oxide) detectors in 3D mode have a well-known limited capacity for acquiring arterial input function using standard 1295 to 1665 MBq (35-45 mCi) bolus Rb-82. Due to this limitation, 3D measures of global LV perfusion are reported using low dose, slow infusion of 700 to 800 MBq Rb-82 (≈ 20mCi or less).^13^ However, this low dose limits statistical content for regional per-pixel perfusion metrics compared to 2D imaging of standard bolus Rb-82.

Current 3D PET scanners use LSO (cerium-doped lutetium oxyorthosilicate) or LYSO (lutetium-yttrium oxyorthosilicate) detectors but for Rb-82 are subject to similar maximum activity limits for arterial input. For dedicated cardiac PET, additional limitations include cost, size, and paucity of extensive clinical outcomes comparable to current literature on 2D PET.

This study has two aims. The first is determining the impact of variable arterial input function that potentially overestimates or underestimates CAD severity leading to either unnecessary interventions or potentially missing beneficial interventions. Analyzing 2D-3D arterial inputs in this study catalyzed the second aim on its capacity for high-activity regional per-pixel perfusion metrics for displaying complex quantitative perfusion per specific artery or branches separately from angiogram that is easily and quickly understood by interventionalists or surgeons not schooled in PET technology or heterogeneous regional coronary physiology for guiding interventions.

## Methods

### Study Population

At Weatherhead PET Center for Preventing and Reversing Atherosclerosis of University of Texas Medical School, written informed consents were obtained from patients with risk factors or clinical indications, referred for diagnostic PET, or volunteers for serial 2D and 3D PET images on separate days, within 2 days to 3 weeks apart. Exclusion criteria included contraindication to dipyridamole, pregnancy, active breastfeeding, clinical instability, and lack of informed consent.

### Cardiac PET Acquisition

Subjects were instructed to fast for 4 hours, abstain from caffeine, theophylline, and cigarettes for at least 24 hours. We used a GE Discovery ST 16 slice PET-CT (Waukesha, Wisconsin), standard bolus infusion of 1100-1850MBq (30-50 mCi) Rb-82 injected at baseline and at 4 minutes after dipyridamole infusion (0.56 mg·kg^−1^) over 4 minutes (0.142 mg·kg^−1^·min^−1^) (Bracco Diagnostics, Princeton, New Jersey) with low-dose CT optimized attenuation co-registration as previously reported.^5^-^12^ Continuous heart rate, blood pressure, and 12-lead electrocardiographic monitoring during stress identified significant, > 1 mm ST-segment depression.

Absolute myocardial perfusion was quantified by HeartSee software (FDA approved 510(k) K171303) using arterial inputs personalized for each PET from among five aortic and left atrium locations^7^ yielding ± 10% test–retest precision in the same patient minutes apart.^6^ Regional per-pixel rest and stress flow (mL·min^−1^·g^−1^), CFR as stress/rest ratio, and coronary flow capacity (CFC) were determined from maximal myocardial activity (and hence statistical certainty) on each of 64 radii in 21 tomogram slices yielding 1344 pixels for LV.^6^-^12^ CFC integrates regional per-pixel values of stress cc·min^−1^·g^−1^ and per-pixel CFR into a clinically defined five color-coded scale (red, orange, yellow, green, and blue) previously reported.^6^-^12^

Different protocols were tested for 2D and 3D acquisition as follows: (i) our standard 2-minute arterial input and 5-minute myocardial image for both rest and during dipyridamole stress validated as equivalent to a compartmental analysis flow model using multiple serial short images for time activity curves to determine perfusion.^4^,^5^ and (ii) a ten-second protocol consisting of 12 ten-second arterial images and 30 ten-second late myocardial images separately reconstructed with corrections for random coincidences, scatter, and dead time loss and summed for 2-minute arterial input and 5-minute myocardial images.

For sensitivity analysis of clinical 2D acquisitions, arterial input changes were simulated in software by changing arterial input by ±10% from original primary clinically measured values in 30 patients. With this ±10% variation of arterial input inserted into the perfusion model, all perfusions metrics were recalculated for all 1344 pixels of LV for each subject in order to determine the effects on recalculated regional CFR and CFC. Statistical differences among perfusion metrics were analyzed by the Kolmogorov–Smirnov test and multivariate bar graphs for statistical difference among 10 studies with normal CFC, 10 studies with intermediate CFC, and 10 studies with severely abnormal CFC.

### Dynamic Circulating Arterial Input Phantom, Volume, and Resolution Phantoms

**Figure 1:**
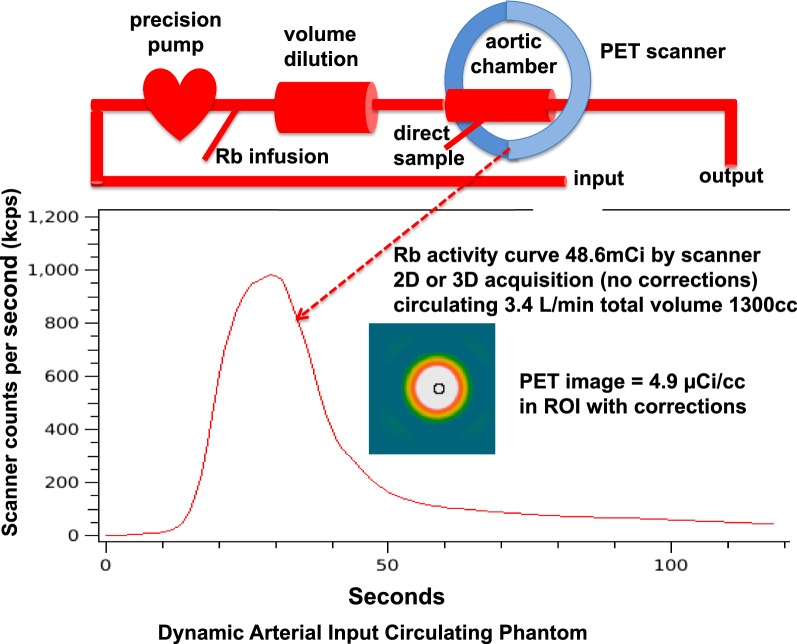
Dynamic circulating model for reproducible “arterial” time activity curves

Since high-count arterial activity images are most susceptible to errors of random coincidences, scatter, and dead time corrections, we tested 3D versus 2D imaging of high-count arterial input using a dynamic circulating arterial phantom (Figure 1). It was constructed of plastic tubing 10 mm internal diameter simulating the aorta with a volume chamber of 1000 mL (18′ long × 2 3/4″ diameter) to simulate the spreading function of lung on arterial activity curves. The system contained 1300 mL of water circulating at 3.4 L·min^−1^ by precision roller pump with injection and sampling ports. Separate input–output buckets provided a continuous loop circulation while avoiding recirculating activity that would warp sharp precisely reproducible arterial activity curves.

However, the circulating phantom does not have surrounding scattering media. In order to assess this additional degrading factor, 3D imaging was done with 2-minute acquisitions of a static 20 cm diameter uniformity phantom containing 5000 mL of water and 999, 666, and 481 MBq (27, 18 or 13 mCi) of Rb-82 providing, respectively, 200, 133, and 96 MBq·mL^−1^ (5.4, 3.6 and 2.6 mCi·mL^−1^). For adequate mixing, the Rb-82 was infused into a 5000 cc water bucket and mixed and poured into the uniformity phantom thereby incurring some decay time during the filling process.

Since high activity of arterial input using standard bolus Rb-82 challenges even advanced 3D PET scanners, we also tested the high-activity capacity and resolution of three 3D GE PET-CT scanners, the DSTE, D710, and DMIc.

### Statistical Analysis

Mean ± standard deviations are reported for continuous variables, number with percent for categorical variables, and median with interquartile range (IQR) for continuous variables with skewed distribution. We utilized paired or unpaired *t* tests to compare continuous variables and Chi-square or Fisher’s exact test to compare categorical data. Kolmogorov–Smirnov (KS) tests compared histogram distributions between groups in color-coded ranges of relative regional uptake images and regional CFC of the LV in previous statistical analysis.^6^

## Results

### Phantom Imaging Tests

**Figure 2:**
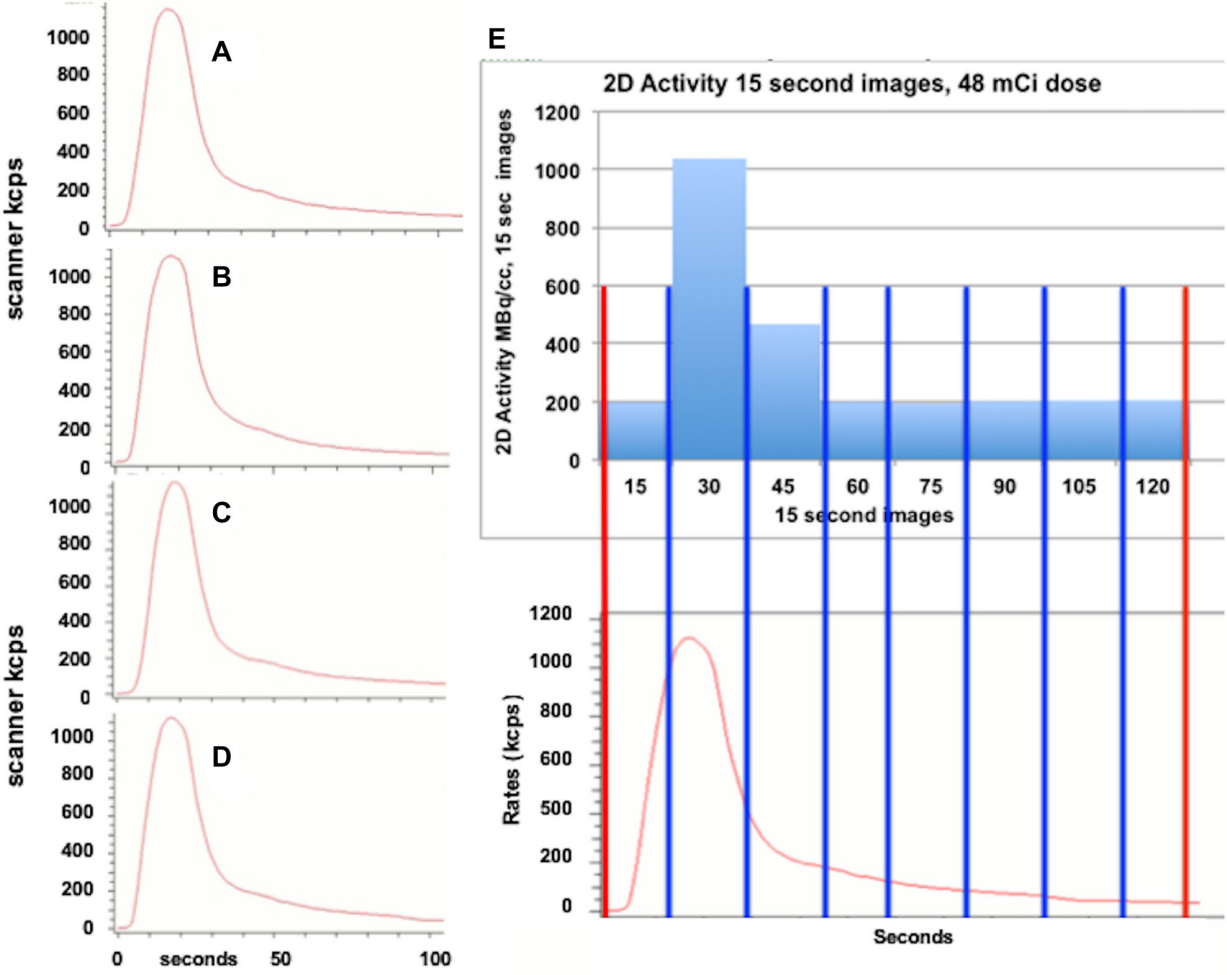
Circulating model arterial time activity curves **A**, **B**, **C,** and **D** shows essentially identical serial repeated total activity–time curves displayed as total counts on the scanner screen after serial separate injections of Rb-82 into the circulating arterial activity model as the input data to the scanner. For each of serial identical time–activity curves as input to the scanner, each scanner protocol was tested for acquiring absolute time activity curves as would be done for the arterial input in patients. Panel E shows the two acquisition protocols—the 2-minute protocol (red brackets) and the serial 15-second (blue brackets) PET acquisition frames with resulting scanner acquired time activity curve. The serial 15-second time–activity was the reference protocol as compared to activity of a timed sample drawn into a syringe during each run after Rb-82 injection and counted in the well counter

Before patient studies, we undertook a series of dynamic circulating phantom studies (Figure 1) in order to design test protocols for 3D imaging in patients. As a first test of scanner capacity to acquire high-count rates in 2D and 3D modes, Figure 2A-D illustrate precise reproducibility of repeated serial total activity curves in the circulating model in real time. For each of serial identical activity curves in the circulating model, two different acquisition protocols in 2D and 3D were compared for quantitative accuracy of scanner integrated time activity.

Figure 2E shows separate scanner acquisitions of the arterial activity curves for these two protocols—serial 15-second frames (blue) and our standard 2-minute arterial image (red). The scanner acquisition frames are decay corrected (also for random coincidences, scatter, and dead time loss) showing the peak and residual activity even though the bolus of activity both decays and is washed out of the open loop circulating model system.

**Figure 3:**
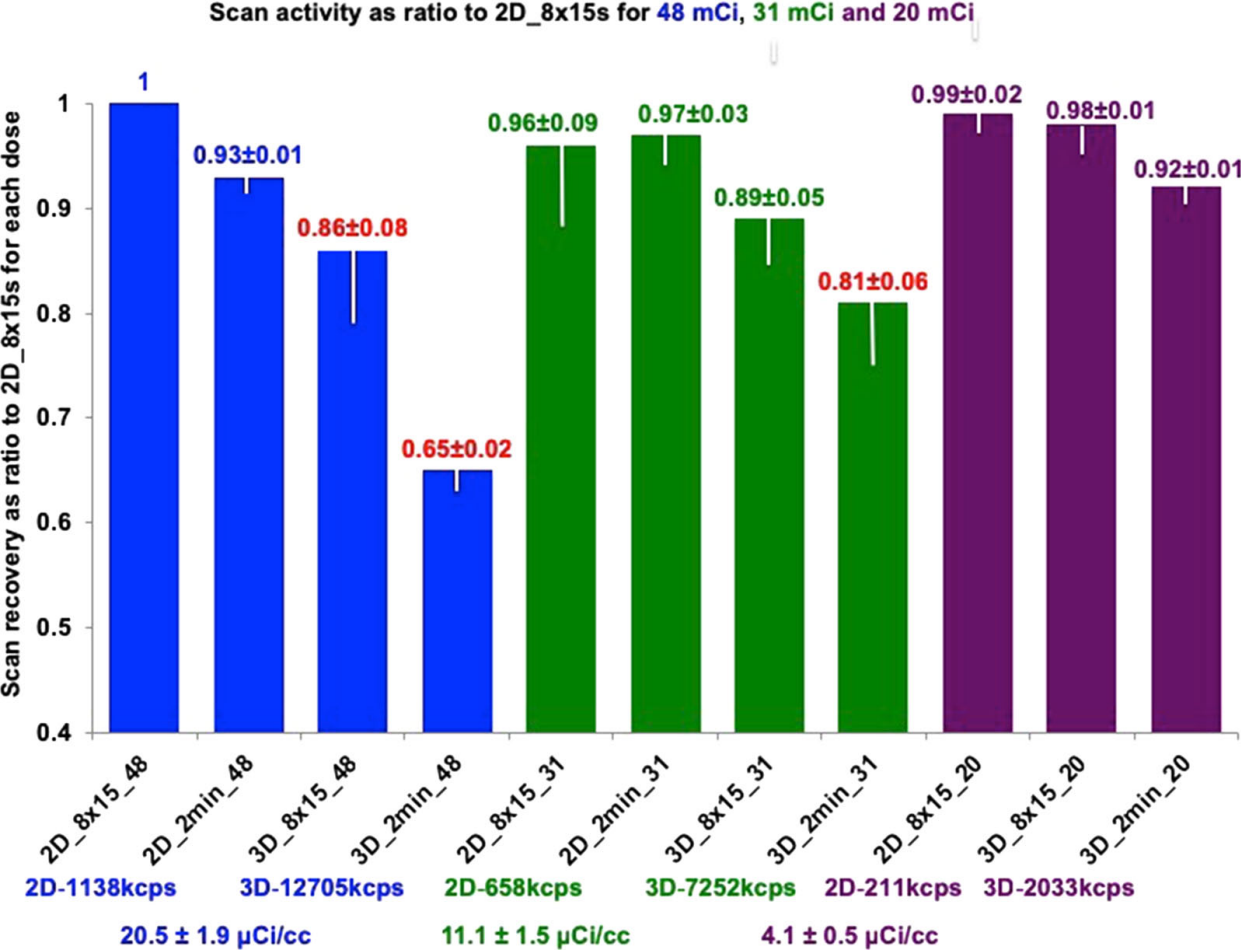
Bar graphs of scanner acquired model arterial activity compared to well counter activity for various doses of Rb-82 with 2D and 3D acquisition. White bars and ± values indicate one standard deviation. The red-highlighted values indicate *P* ≤ 0.005 compared to the highest value for the Rb-82 dose coded blue 1176 MBq (48 mCi), green 1147 MBq (31 mCi), or purple 740 MBq (20 mCi)

Figure 3 summarizes 2D or 3D scanner arterial input function for serial identical arterial activity curves of the dynamic circulating phantom for up to 1295-1480 MBq (35-40 mCi) Rb-82 injected into the circulating model expressed as a ratio to the arterial input function of the ideal most accurate 2D protocol comprised of eight serial 15-second images in the first bar of the graph. Peak activities by the scanner for each dose and acquisition protocol are listed below the bar graph with arterial input function in µCI·mL^−1^ of a 30 to 50 mL sample drawn during circulating high arterial activity and counted in a dose calibrator (DC). By dose calibrator, activities of the circulating model samples ranged from 152 ± 19 (4.1 ± 0.5) to 759 ± 70 MBq·mL^−1^ (20.5 ± 1.9 µCi·mL^−1^).

The data indicate that 2D accurately measures the arterial input function over the range of low- to high-dose Rb-82 with serial 15-second or single 2-minute acquisition protocols. Scanner arterial activity for 1776 MBq (48 mCI) injected into the circulating model is slightly, insignificantly underestimated at the highest activity concentrations of 740 MBq·mL^−1^ (20 µCi·mL^−1^) approximating those seen clinically after 1480 MBq (40 mCi) Rb-82.

At high doses of 1776 MBq (48 mCi) Rb-82, the 3D acquisition significantly underestimates the arterial input function using either serial 15-second or single 2-minute acquisitions. At a dose of 1147 MBq (31 mCi), the 3D single 2-minute acquisition significantly underestimates arterial input. However, the 3D serial 15-second acquisition approximates the arterial input with a statistically insignificant modest underestimation. At the 740 MBq (20 mCi) dose, 3D acquisition measures the arterial input using both the serial 15-second and single 2-minute acquisition protocols.

The data from the dynamic circulating arterial phantom test the 3D capacity for random coincidences and dead time loss over a range of Rb-82 doses and acquisition protocols without surrounding scattering media and therefore do not test added scatter correction. Accordingly, 3D imaging was done with 2-minute acquisition of a static 20 cm diameter uniformity volume phantom containing 5000 cc filled with water and 999, 666, and 481 MBq (27, 18 or 13 mCi) of Rb-82 providing, respectively, 200, 133, and 96 MBq·mL^−1^ (5.4, 3.6 and 2.6 mCi·mL^−1^). For 3D acquisition, the dose of 999 MBq (27 mCi) in this uniformity phantom caused severe ring artifacts that precluded useful images reconstruction. For 3D acquisition at the dose of 666 (18) and 481 MBq (13 mCi), images could be reconstructed but had visible ring artifacts precluding quantification. Thus, with scattering media at these activity concentrations, 3D imaging with a 2-minute acquisition protocol did not acquire adequate images despite imaging adequate arterial input for comparable activity concentrations in the absence of scattering media.

### Effects of ± 10% change in arterial input on CFR

**Figure 4:**
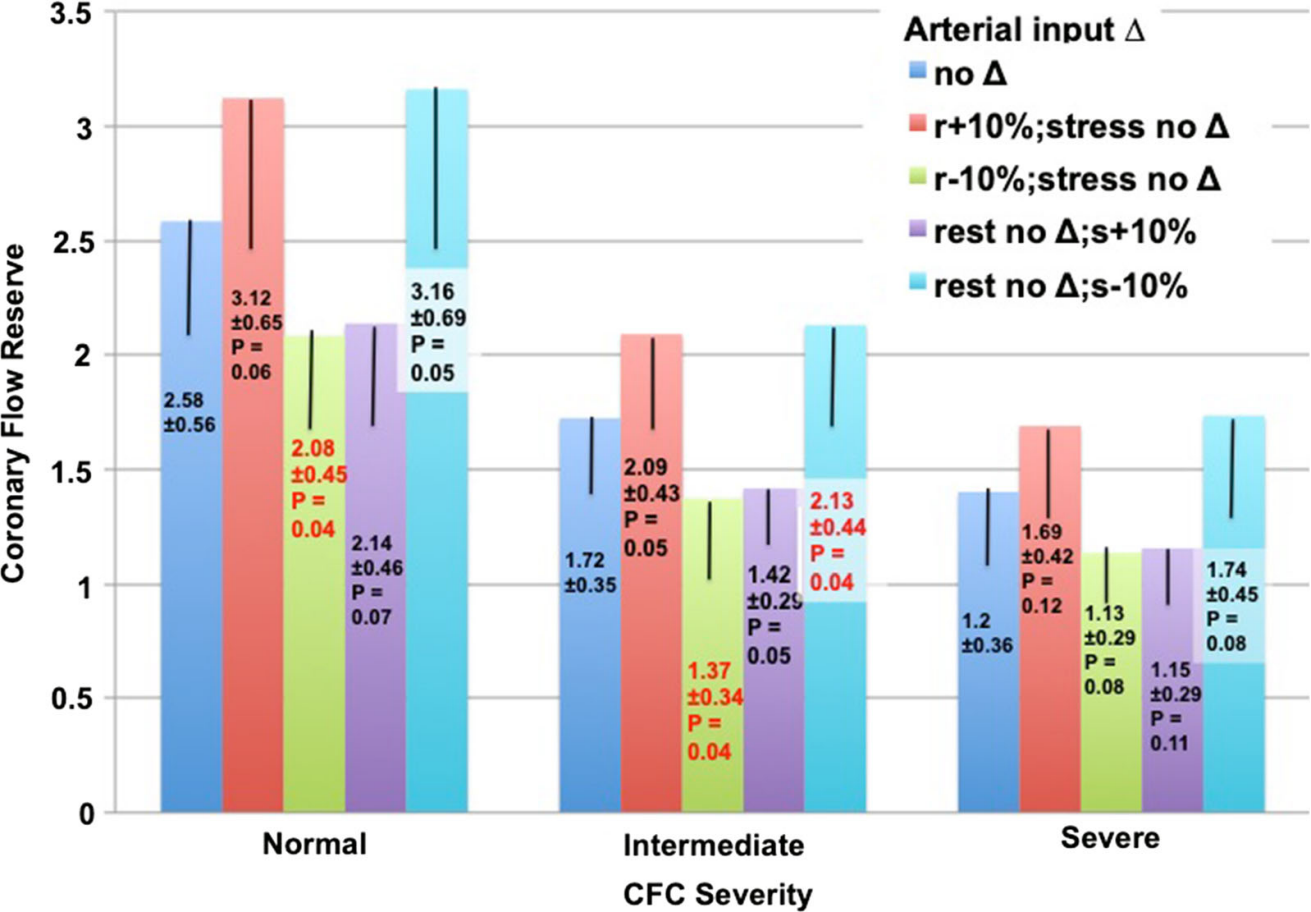
Effect on coronary flow reserve of rest and stress arterial activity increased or decreased by 10% compared to the standard optimal arterial activity in 30 subjects in each of three representative groups—normal or mildly reduced, intermediate, or worst severity of coronary flow capacity (CFC) that accounts for both rest–stress perfusion in cc·min^−1^·g^−1^ and CFR. Vertical bars and ± values indicate one standard deviation. Red-highlighted data indicate significance with *P* < 0.05

In Figure 4, increasing arterial input in software by 10% over measured rest arterial input lowered rest perfusion that increased CFR and for the stress arterial input lowered stress perfusion that lowered CFR. Decreasing arterial input by 10% below measured rest arterial input raised rest perfusion that lowered CFR and for the stress arterial input increased stress perfusion that increased CFR. Increasing or decreasing arterial input for both rest and stress perfusion in parallel had little effect on CFR whereas changing arterial input in opposite directions for rest and stress had more prominent changes than for rest or stress alone.

For normal relative stress images or for severely abnormal relative stress images, ± 10% change in arterial input had little clinically significant impact on CFR that remained over 2.0 or severely reduced below 2. However, for intermediate severity, + 10% change in arterial input for rest perfusion increased CFR to over 2 compared to CFR of 1.72 with measured arterial input. Similarly, a (−)10% change in arterial input for stress perfusion also increased CFR to over 2.

### Effects of ± 10% change in arterial input on CFC by Kolmogorov–Smirnov test

**Figure 5:**
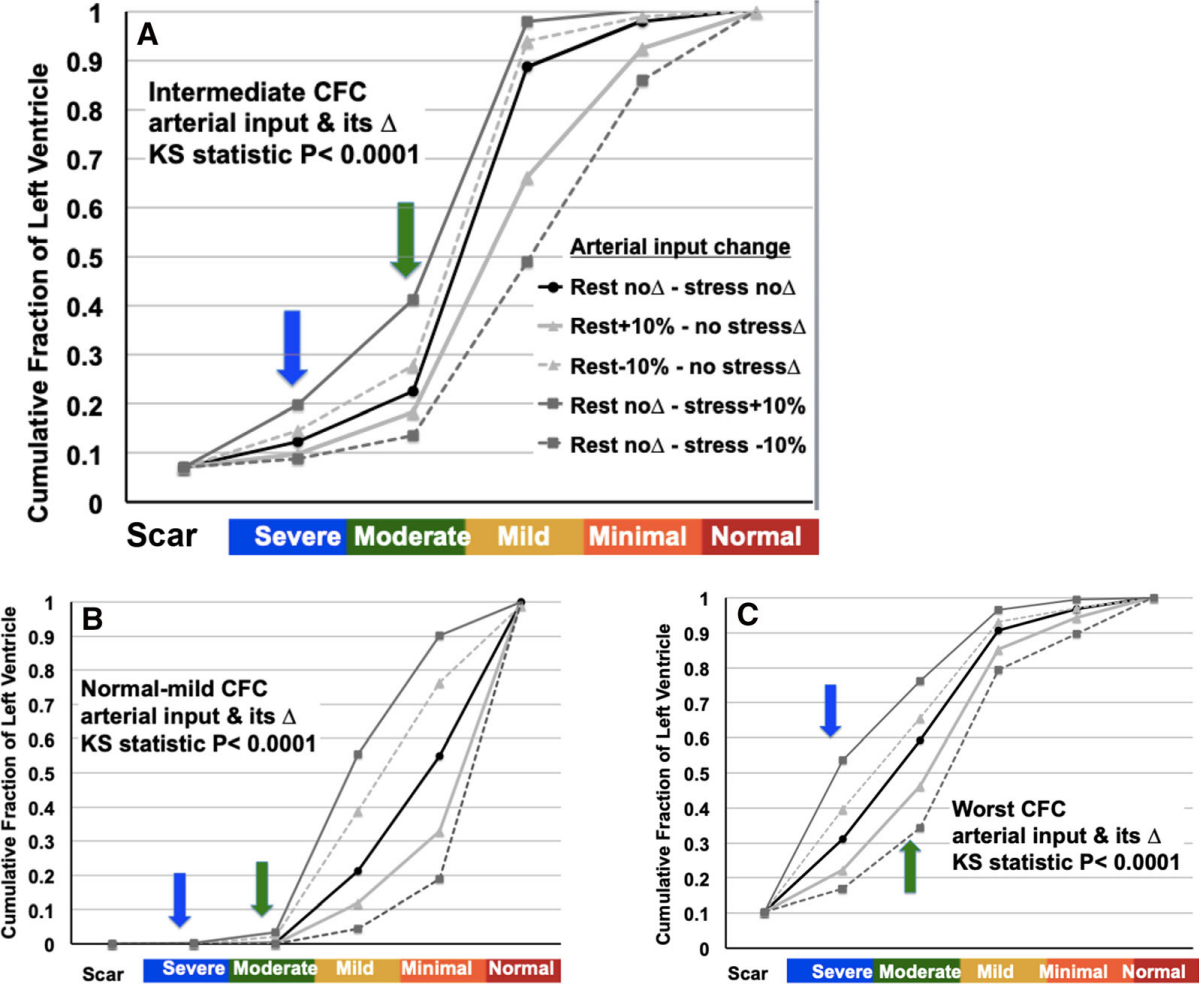
Cumulative fraction of LV in CFC severity ranges and Kolomogorov–Smirnov statistics for significant differences in histogram distributions with rest and stress perfusion increased or decreased by ±10% (gray lines) versus no change (black line) for 10 patients in each of the following groups: (**A**) intermediate CFC abnormalities; (**B**) normal or mildly reduced CFC; (**C**) worst CFC abnormalities. The KS statistic for all gray lines versus the black line is significant with *P* ≤ 0.0001. The blue and green arrows emphasize the changes in moderate to severe CFC abnormalities having greatest impact on potential interventions based on CFC severity

For mild, intermediate, and severely reduced CFR severities, the arterial input function was changed by (+) and (−) 10% from our standard clinical PV correction and all perfusion metrics for all pixels were recalculated in automated software. For intermediate CFC severity, the ± 10% change in arterial input function shifted the distribution of moderately to severely reduced pixels and of small to large or vice versa that comprised potentially significant changes to above or below clinically relevant severity threshold for guiding interventions (Figure 5A green and blue arrows).

For normal or mildly reduced CFC maps, a ± 10% change in arterial input from our standard clinical PV corrections shifted the distribution of normal and mildly reduced pixels even more towards normal or caused only minimal clinically insignificant worsening thereby indicating no clinically significant change for or against severity altering interventional decisions (Figure 5B green and blue arrows). For severely reduced CFC maps, ± 10% change in arterial input shifted the distribution of moderately and severely reduced pixels that remained substantially abnormal, hence still indicating potential intervention (Figure 5C green and blue arrows).

Table 1 quantifies these the shift in the histogram distribution of pixel severities for the various combinations of ± 10% change in arterial input. For the 3987 rest–stress serial diagnostic PETs in this study, 7% of 3774 PETs fell into this intermediate category, 19% fell into the severe category and 44% fell into the mild to normal category.

**Table 1:** Percent of LV with moderate or severe CFC pixels for intermediate and severe population with ±10% change in arterial input

Population group	CFC pixel severity	Nor stress/nor rest	Rest + 10%/nor strs	Rest − 10%/nor strs	Nor rest/strs + 10%	Nor rest/strs − 10%
Inter mediate	Moderate	10.3±10.1%	8.5±9.7%	13.3±8.5%	21.6±7.4%	4.6±5.2%
	Severe	5.3±6.5%	2.7±3.9%	7.4±8.0%	12.7±13.2%	1.8±2.9%
Severe	Moderate	28.2±14.5%	24.0±12.4%	26.1±12.3%	22.4±10.5%	17.3±8.3%
	Severe	20.8±9.6%	11.8±9.6%	29.3±13.6%	43.3±19.9%	6.5±5.2%

### Arterial Input, 3D versus 2D imaging and regional pixel activity

**Figure 6:**
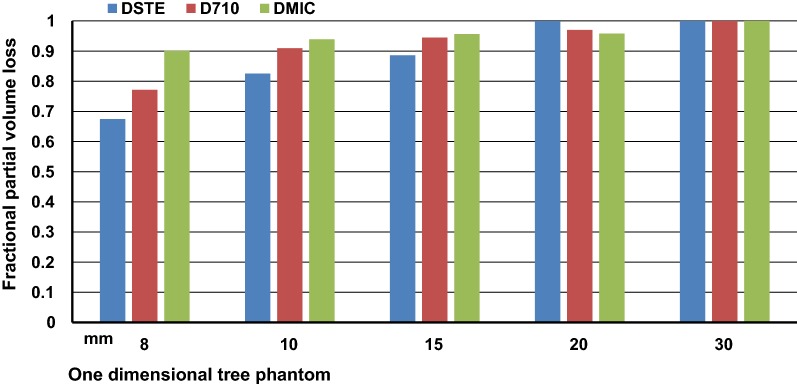
Fractional partial volume activity loss for 8 to 30 mm wide one-dimensional tree phantom imaged by GE DSTE, D710, and DMic PET-CT scanners

Compared to 2D, current 3D scanners using LYSO or LSO detectors offer improved resolution and sensitivity for acquiring more true coincidence counts for a given dose as in Figure 6. However, quantitative activity recovery deteriorates at high-activity concentrations for 3D (Table 2) as evident by random coincidences being higher than true coincident counts with 1.7 random for every true coincidence count. However, this 3D high-activity capacity opens two subtle but distinctly different paths for different perfusion models, how the flow data are used and their clinical applications in CAD as follows.

**Table 2:** Activity recovery for concentrations of F-18 and PET-CT scanners in 500 mL

	µCi·mL^−1^	SUV	Total (M)	Trues/randoms	Ratio true/rand
DSTE—2D (4.3 mCi/500 mL) DSTE—3D (4.1 mCi/500 mL	8.5 8.2	1.0 0.95	243 1156	217 (true + rand)/1027	
D710—3D (4 mCi/500 mL) D710—3D (18.3 mCi/500 mL)	7.9 37	0.94 0.84	988 5273	661/218 1855/3147	3 to 1 0.6 to 1
DMI—3D (4.2 mCi/500 mL)	8.3	0.89	2580	1356/909	1.5 to 1

**Figure 7:**
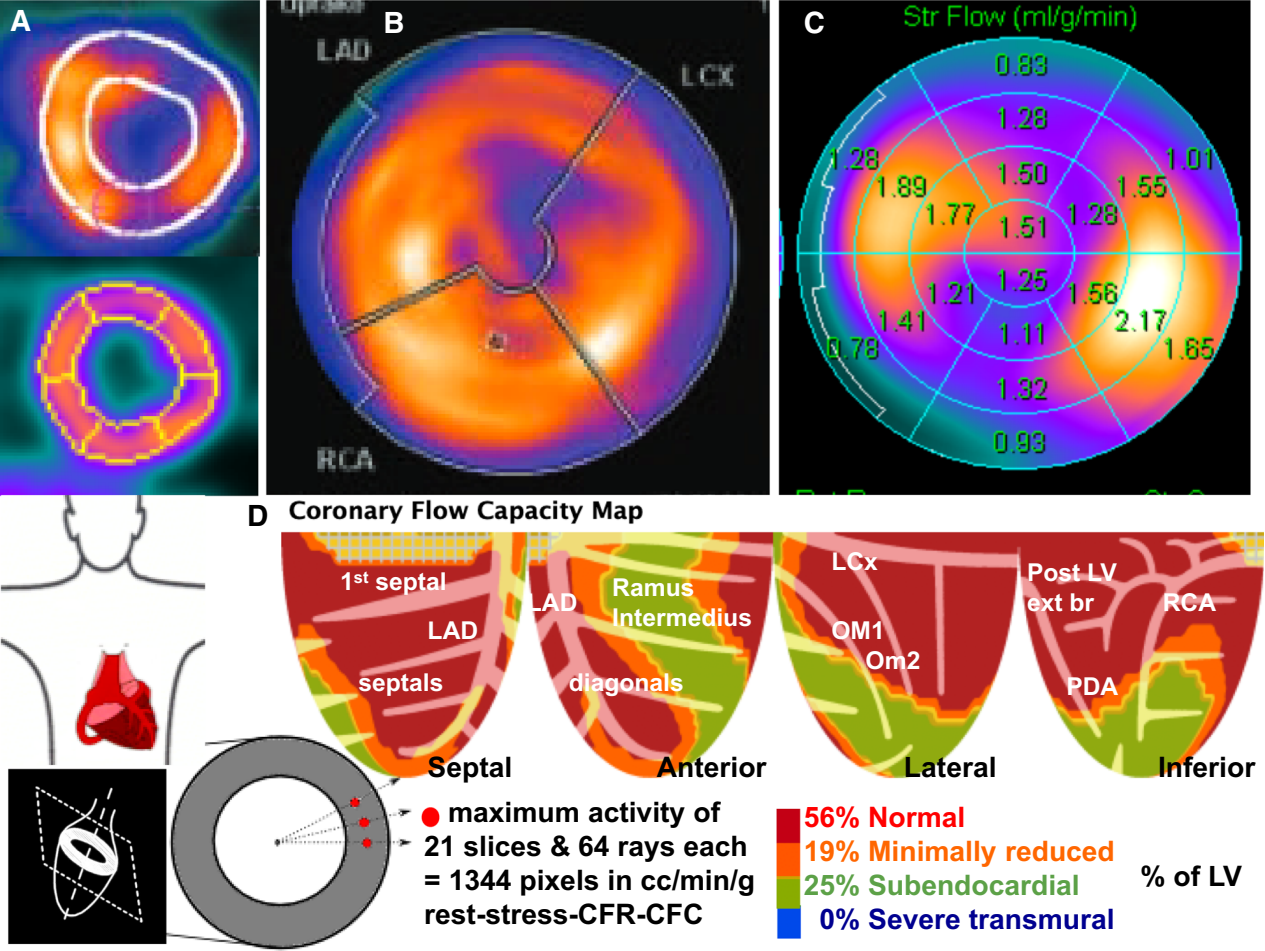
Three types of Regions of Interest (ROI) or regional boundaries in which quantitative metrics are measured ranging from large externally imposed, fixed whole artery distributions (**A** and **B**) to fixed external multi-segmental compartments (**C**) to per-pixel values (**D**) providing perfusion, CFR, and coronary flow capacity (CFC) maps of precise, actual perfusion arterial distributions for each individual as they actually are without assumed arbitrary fixed externally imposed regions of interest for which average perfusion is determined

In Figure 7A and B, quantitative perfusion is measured within large areas of LV corresponding to the average, fixed, externally imposed assumed distributions of the three major coronary arteries and bounded by endocardial–epicardial borders. Since this analysis and display averages data over large areas, it does not require high statistical counts per-pixel thereby allowing very low dose of Rb-82 for visual interpretation and average perfusion in large predefined regions. However, it does not show size and severity of specific perfusion anatomic distribution of coronary arteries or branches as they actually are.

For a different patient, the perfusion model and display in Figure 7C has 21 fixed externally imposed regions to quantify perfusion in more granular regional distribution. It is clearly abnormal but quantitative size-severity for what artery or arteries are not sufficiently defined for this patient’s cardiologist to decide on invasive or non-invasive management. This approach also averages data within fixed, externally imposed, assumed segments comprising one 21st of the LV that does not define arterial perfusion anatomy actually present. However, this approach is the most widely used, provides adequate images for visual and approximate perfusion at low-dose Rb-82.

In order to make an informed clinical decision, the cardiologist sent this patient for PET using an alternative perfusion model and analytical display in Figure 7D. This perfusion model determines per-pixel maximal myocardial activity with greatest statistical certainty for each of 64 radii of 21 slices for 1344 pixels of the LV each with per-pixel values of rest, stress cc·min^−1^·g^−1^, coronary flow reserve (CFR), and coronary flow capacity (CFC). CFC combines stress perfusion and CFR for each pixel to account for variable perfusion heterogeneity due to endothelial dysfunction or risk factors. This very large data set is objectively and physiologically compressed into a clinically defined color-coded map for ranges of combined stress perfusion and CFR as previously reported.^6^,^11^,^12^ The CFC maps show exact actual perfusion arterial distributions for each individual as they actually are in a display similar to cardiologist’s views of angiograms without the assumed externally imposed arterial distributions or the spatial anatomic distortion caused by the bull’s eye display.

From the CFC map, the cardiologist readily recognized stenosis of a Ramus Intermedius branch with low-risk, mildly reduced subendocardial/subepicardial perfusion ratio but with adequate absolute perfusion without ischemia in addition to excellent flow in all other coronary arteries, best treated medically. That decision was confirmed by repeat PET 2 years later showing substantial improvement as an example of low-risk CAD reported systematically for large cohorts.^1^,^2^

**Figure 8:**
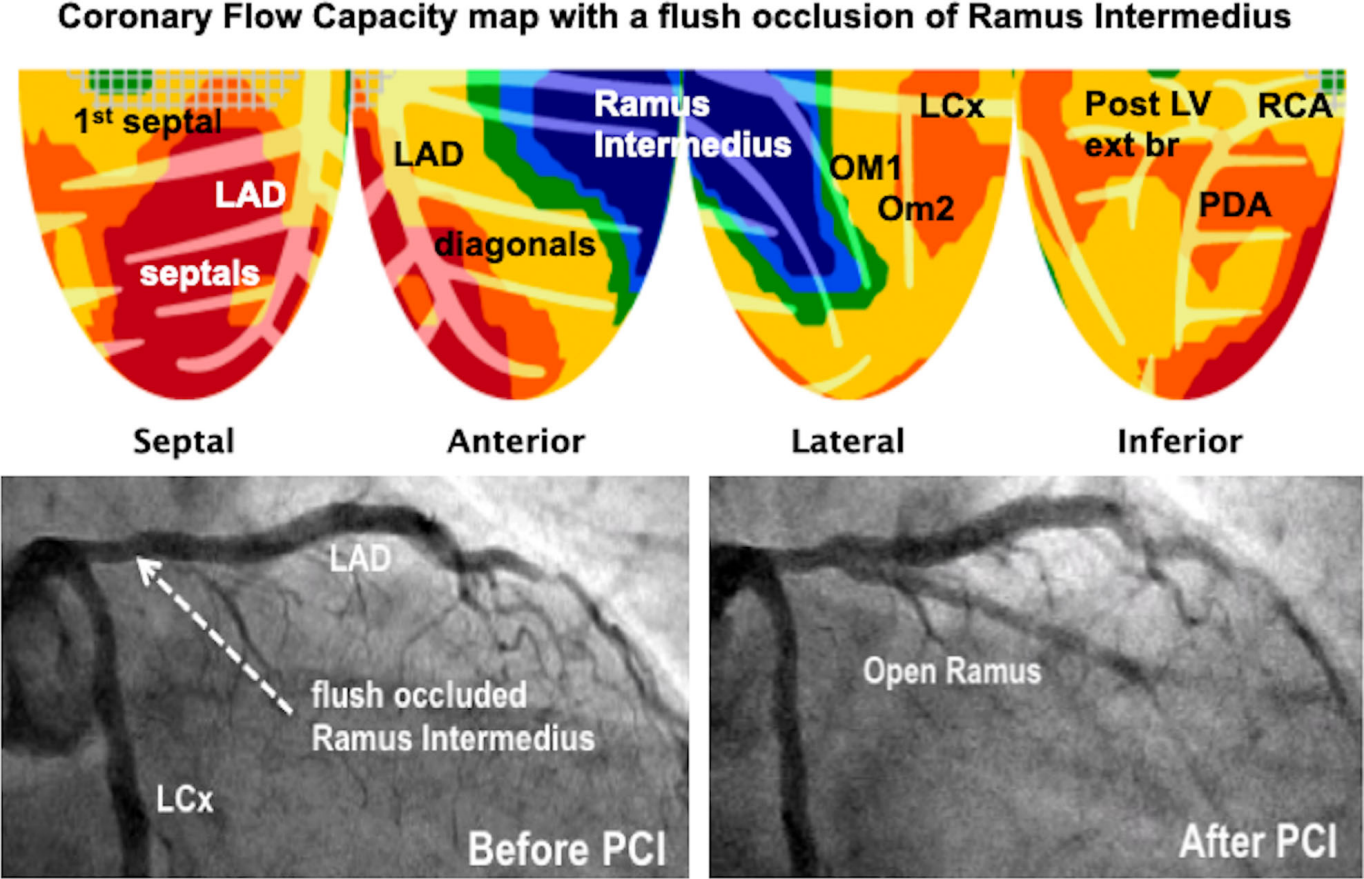
Even angiogram anatomy does not define how regions of interest should be drawn for regional perfusion measurement

Even invasive angiogram or non-invasive CTA providing precise anatomy does not resolve how regions of interest should be drawn for quantifying perfusion as done in Figures 7B and C. In Figure 8, angiogram shows no stenosis to explain angina. However, per-pixel quantification reveals true size-severity of perfusion anatomy due to flush occlusion of a large Ramus Intermedius with myocardial steal indicating collaterals to viable myocardium, a not uncommon finding for stent jailed branches.

This perfusion model using per-pixel distribution to define actual perfusion anatomy is *most precise for high per-pixel activity* after 1110-1665 MBq (30-45 mCi) of Rb-82 with 2D imaging or at whatever lesser dose 3D PET-CT can acquire. The patient radiation exposure for 740 MBq (20 mCi) compared to 1665 MBq (45 mCi) of Rb-82 is approximately one millisievert balanced against the clinical value of quantitative size-severity perfusion anatomy to guide invasive procedures having substantially greater risk of an unnecessary procedure or the risk of withholding it.

## Discussion

Our results suggest that ± 10% variability of arterial input function has little impact on less severe abnormalities of stress perfusion, CFR or CFC for which angiogram or interventions are unlikely. Similarly, the effect of ± 10% variability in arterial input function on severe perfusion abnormalities did not alter their indication for an angiogram or intervention based on PET severity. However, for intermediate, border zone severity of stress perfusion, CFR and CFC, arterial input variability of ± 10% may cause over or underestimation of severity leading to unnecessary, or potentially missing, beneficial interventions important for optimized individualized management.

For 3987 rest–stress serial diagnostic PETs in this study, 7% fell into this intermediate category, 19% fell into the severe category, and 44% fell into the mild to normal category. Therefore, for this small but highly individually relevant group, critical analysis of arterial input function is essential for optimal individualized management where high-quality arterial phase images are essential. While other scanners and protocols may have different thresholds at which ± 10% may be important, the principle we report applies to all PET scanners for sites using PET to guide coronary procedures whereby interventionalists readily understand and “own” their patients' perfusion data.

Our secondary aim in this study identified cumulative failure of corrections for random coincidence, dead time, and scatter loss with 3D BGO imaging at increasing dose of Rb-82. As detailed in the Supplemental, the 3D images were substantially improved by acquiring short 10-second images with separate random, dead time, and scatter corrections that are summed for a 2-minute arterial input or 5-minute myocardial image appropriate for the retention perfusion model. Since current 3D PET-CT incurs cost and build-out space limiting widespread dedicated cardiac PET, these observations may suggest potential design modification for low-cost, small, dedicated cardiac PET using BGO detectors until current 3D systems evolve for less costly dedicated cardiac application.

## Limitation of Findings

While our results are derived from an established 2D/3D BGO PET-CT like those used for the great majority of quantitative myocardial perfusion literature, the principles we report apply to 3D LSO, LYSO, or solid state scanners for bolus Rb-82 since these scanners were designed for lower-dose F-18 or N-13 and lack extensive literature documenting clinical application of quantitative perfusion using standard bolus Rb-82 perfusion comparable to the extensive literature from 2D BGO PET scanners.

## New Knowledge Gained

While quantitative myocardial perfusion by 2D PET predicts high-risk CAD that is significantly reduced by revascularization, 3D PET using Rb-82 for arterial input and quantitative myocardial perfusion requires cardiac specific different acquisition protocols than 2D PET that need validation in comparison with outcomes after PET-guided interventions.

## Conclusions

For intermediate, border zone severity of stress perfusion, CFR and CFC comprising 7% of 3987 cases, an arterial input variability of ±10% may cause over or underestimation of severity leading to unnecessary interventions or potentially missing beneficial interventions essential for optimal individualized management. Current 3D PET-CT with LYSO or LSO detectors appears suitable for quantitative perfusion using standard bolus Rb-82 but is not yet validated comparably to the large clinical literature on 2D cardiac PET. However, 3D PET adds potential of high-activity per-pixel values of mL·min^−1^·g^−1^ for rest, stress, CFR, and CFC to define precisely quantitative perfusion anatomy familiar to interventionslists for guiding invasive procedures.

## Electronic supplementary material

Below is the link to the electronic supplementary material.

Electronic supplementary material 1 (DOC 482 kb)

Electronic supplementary material 2 (PPTX 14870 kb)

Electronic supplementary material 3 (PPTX 259 kb)

Electronic supplementary material 4 (M4A 3077 kb)

## Abbreviations

BGO: Bismuth germanium oxide or bismuth germanate
CAD: Coronary artery disease
PET: Positron emission tomography
CFR: Coronary flow reserve
SD: Standard deviation
PET/CT: Positron emission tomography—computed tomography
KS: Kolmogorov–Smirnov statistic
LV: Left ventricular
ROI: Region of interest
Rb-82: Rubidiun-82
