# Supplementary material for: Pitfalls in quantitative myocardial PET perfusion II: Arterial input function
Source: J Nucl Cardiol. 2020 Mar 3;27(2):397–409. doi: 10.1007/s12350-020-02074-8 (PMC7174279; doi:10.1007/s12350-020-02074-8)

**On Line Resource - Supplement JNC 10-23-19**

**2D versus 3D in patients using a 2D/3D PET-CT scanner with BGO detectors using a modified 3D acquisition protocol for Rb-82.** Serial 3D and 2D rest-stress imaging in 22 patients using our standard 2 minute arterial acquisition and a range of Rb-82 doses showed severe, large artifacts on 3D images compared to normal 2D images for the same patient. Due to frequent artifacts causing severe large perfusion defects on 3D images not seen on 2D images, the histogram distribution of relative activity of 3D images [**Figure OR1**] (blue line with squares) was significantly shifted leftward toward greater relative severity compared to 2D images (red solid line with circles). The Kolmogorov – Smirnov (KS) statistic for differences in the histogram distribution of myocardial relative activity between 3D and 2D images is highly significant.

We then tested the hypothesis that the mechanism for artifacts in 3D images using the 2 minute arterial acquisition was due to inadequate corrections for random coincidences, scatter and dead time loss over the entire 2 minute protocol due to high counts in the 3D images compared to 2D images. Based on the 15 second serial image acquisition data of the dynamic circulating arterial phantom, the rest – stress myocardial images in patients were obtained as serial 10 second images reconstructed separately with corrections for random coincidences, scatter, and dead time loss. These individually corrected 10 second images were then summed to provide a single 2 minute arterial input function and 5 minute myocardial images for our retention flow model. These 3D serial, summed arterial and myocardial images were more comparable to 2D data but with some remaining artifacts.

The average cumulative relative activity distribution on the KS plot shifted rightward to match the 2D data [**Figure OR1**] (red dashed line with triangles). However, for individual PETs, the regional pixel quantification was heterogeneously inferior to 2D images. Moreover, reconstructing the forty-two serial 10-second images for rest and for stress images required over an hour for each 3D rest stress PET study. This substantial but incomplete improvement suggests a potential direction for possible design modifications that might make possible low cost, small, BGO PET-CT for dedicated cardiac PET using standard bolus Rb-82.


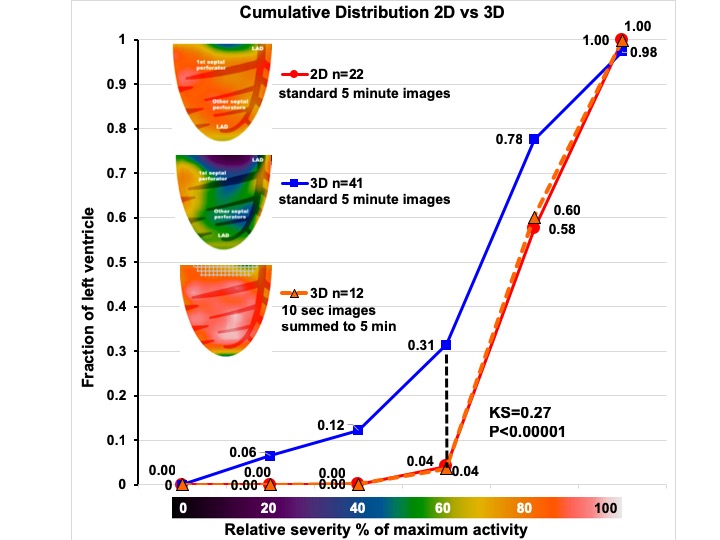


**Legend for On Line Resource Figure OR1**

Comparison of relative images by 2D versus 3D acquisition for 5-minute myocardial acquisition and for 3D serial 10 second acquisitions separately reconstructed with corrections for random coincidences, scatter and dead time losses, then summed to 5-minute images with the Kolmogorov – Smirnov (KS) statistic for differences in cumulative histogram distributions of relative activity throughout the LV.

**Legend for On Line Resource Figure OR2**

Single quadrant views of relative activity for every PET for which 3D acquisition could be reconstructed despite obvious artifactual images not seen on 2D PET of the same subject. Of serial 2D and 3D PETs attempted in 22 subjects, 3D PET images could not be reconstructed in 10 (45%). Eleven (50%) had images with significant visual artifacts not present on 2D images [**Figure OR2 (A) and (B)**] and one (5%) had images comparable to 2D [**Figure OR2 (C)**]. In order to document limitations of 3D for acquiring high activities on the BGO scanner as due to activity not radionuclide, we also used N-13 ammonia with results similar to Rb-82 [Figure OR2]. The extent of artifactual images was only approximately related to upper half or lower half of dose ranges.

**Figure OR2 (A)**


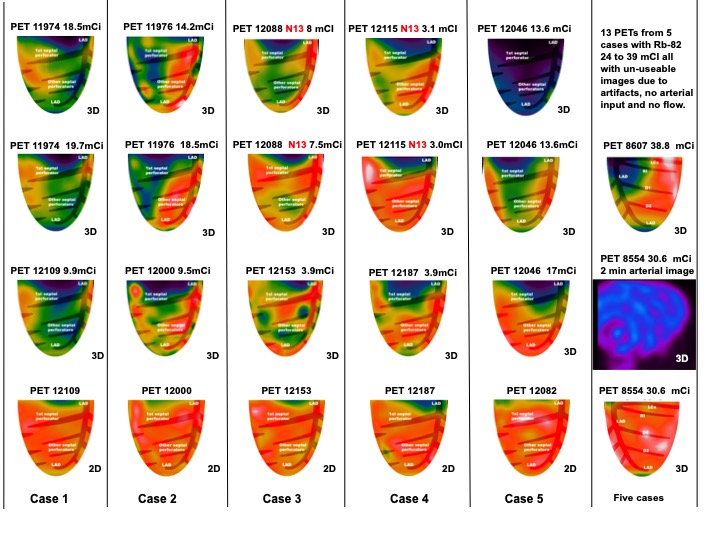


**Figure OR2 (B)**


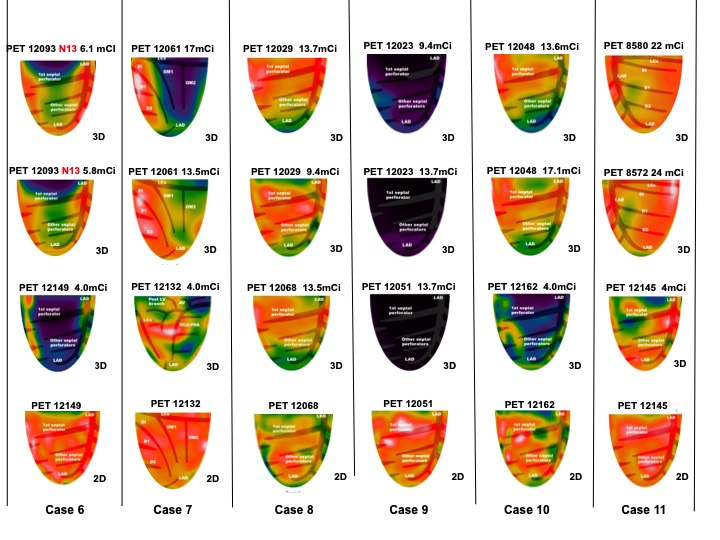


Of the 22 cases for which 3D acquisition was attempted on the BGO scanner, only one case [**Figure OR2 (C)**] had adequate 3D acquisition by summed serial 10 second corrected images of stress relative images (top row), stress cc/min/g (middle row) and CFC map combining per-pixel stress perfusion, that were comparable to 2D quantitative PET. For this case, the Rb-82 dose was 873 MBq (23.6 mCi) and the patient weighed 270 lbs at 6 feet tall (BMI 37).


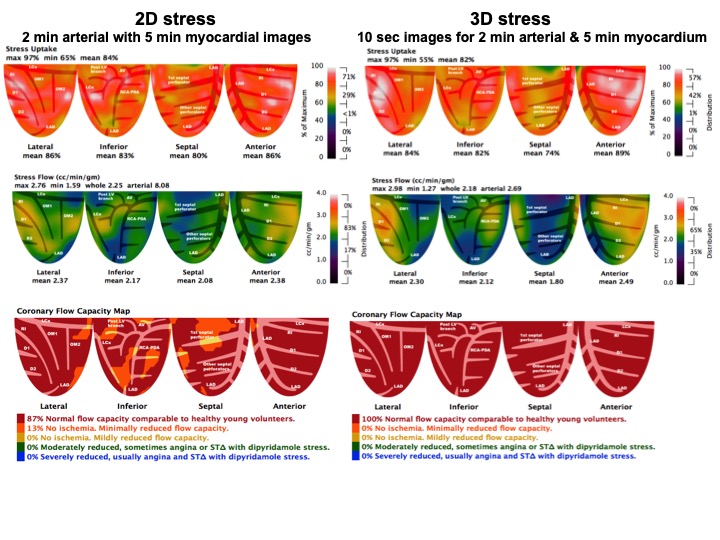

Supplement: Supplementary file 1 — Electronic supplementary material 1 (DOC 482 kb) [file 12350_2020_2074_MOESM1_ESM.doc]
