# Supplementary material for: Pitfalls in quantitative myocardial PET perfusion II: Arterial input function
Source: J Nucl Cardiol. 2020 Mar 3;27(2):397–409. doi: 10.1007/s12350-020-02074-8 (PMC7174279; doi:10.1007/s12350-020-02074-8)
Supplement: Supplementary file 2 — Electronic supplementary material 2 (PPTX 14870 kb) [file 12350_2020_2074_MOESM2_ESM.pptx]

## Slide 1
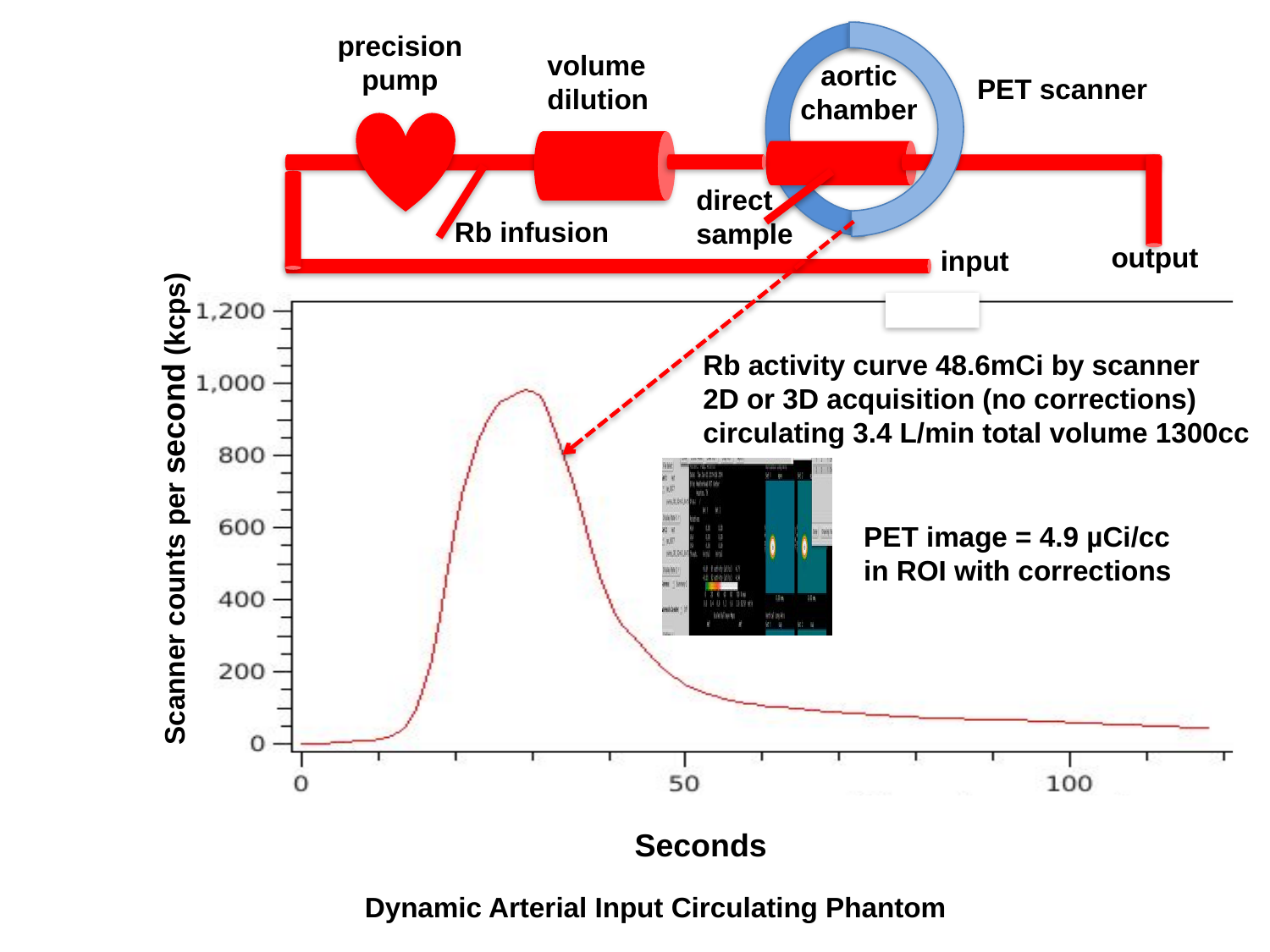

precision
pump
volume
dilution
aortic
chamber
PET scanner
direct
sample
Rb infusion
output
input
Rb activity curve 48.6mCi by scanner
2D or 3D acquisition (no corrections)
circulating 3.4 L/min total volume 1300cc
Scanner counts per second (kcps)
PET image = 4.9 µCi/cc
in ROI with corrections
Seconds
Dynamic Arterial Input Circulating Phantom

## Slide 2
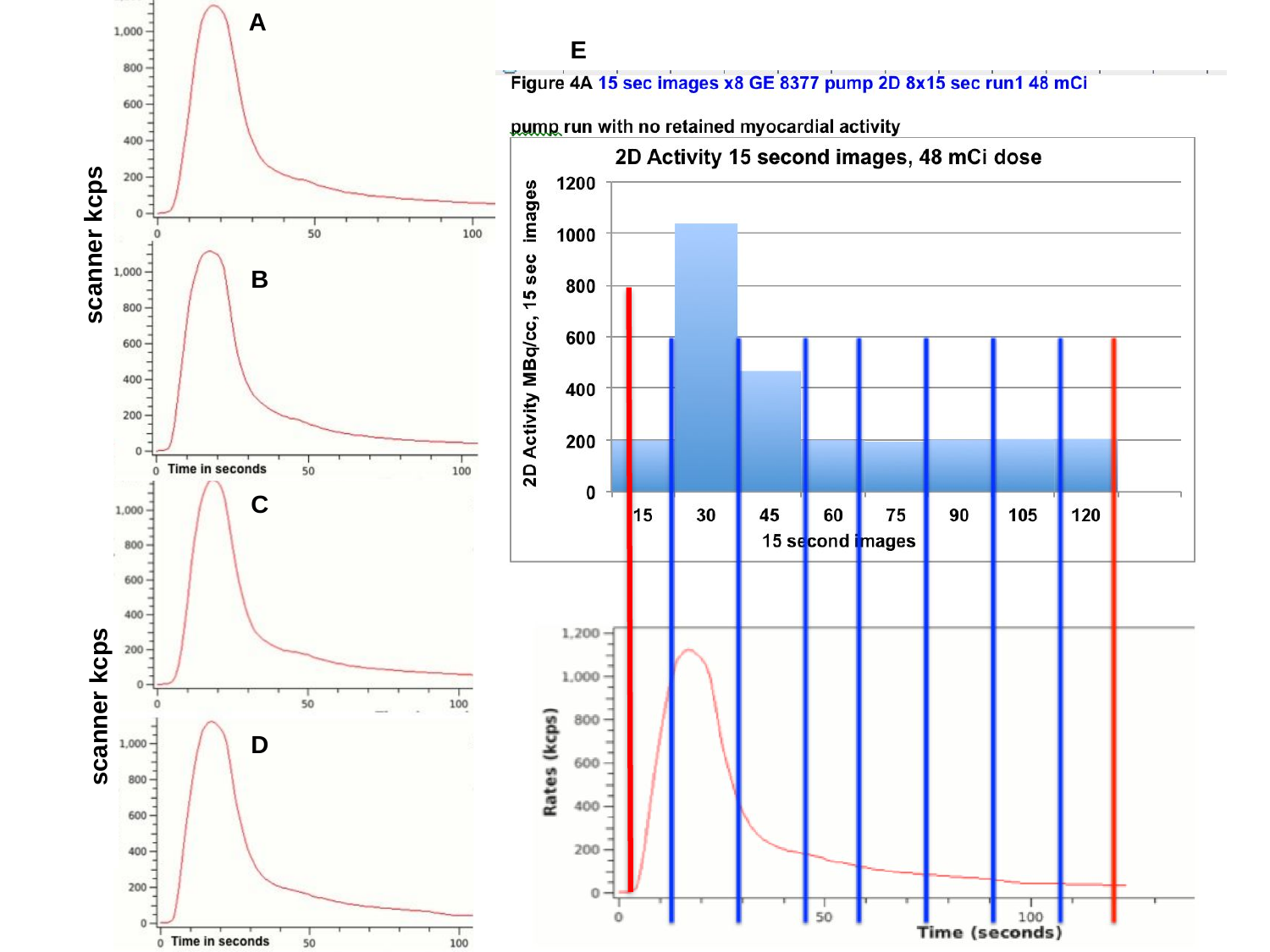

A
E
scanner kcps
B
C
 scanner kcps
D

## Slide 3
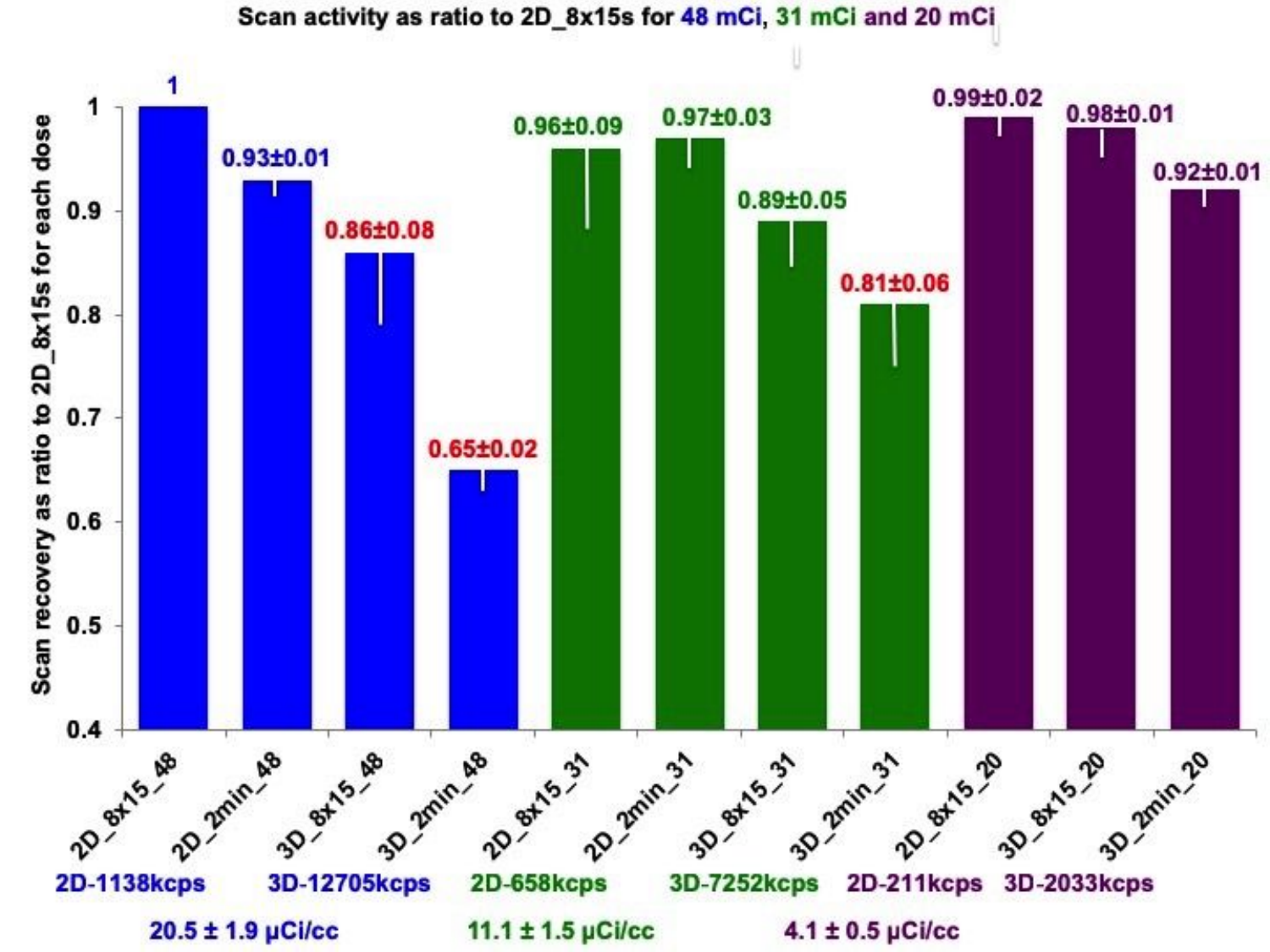

## Slide 4
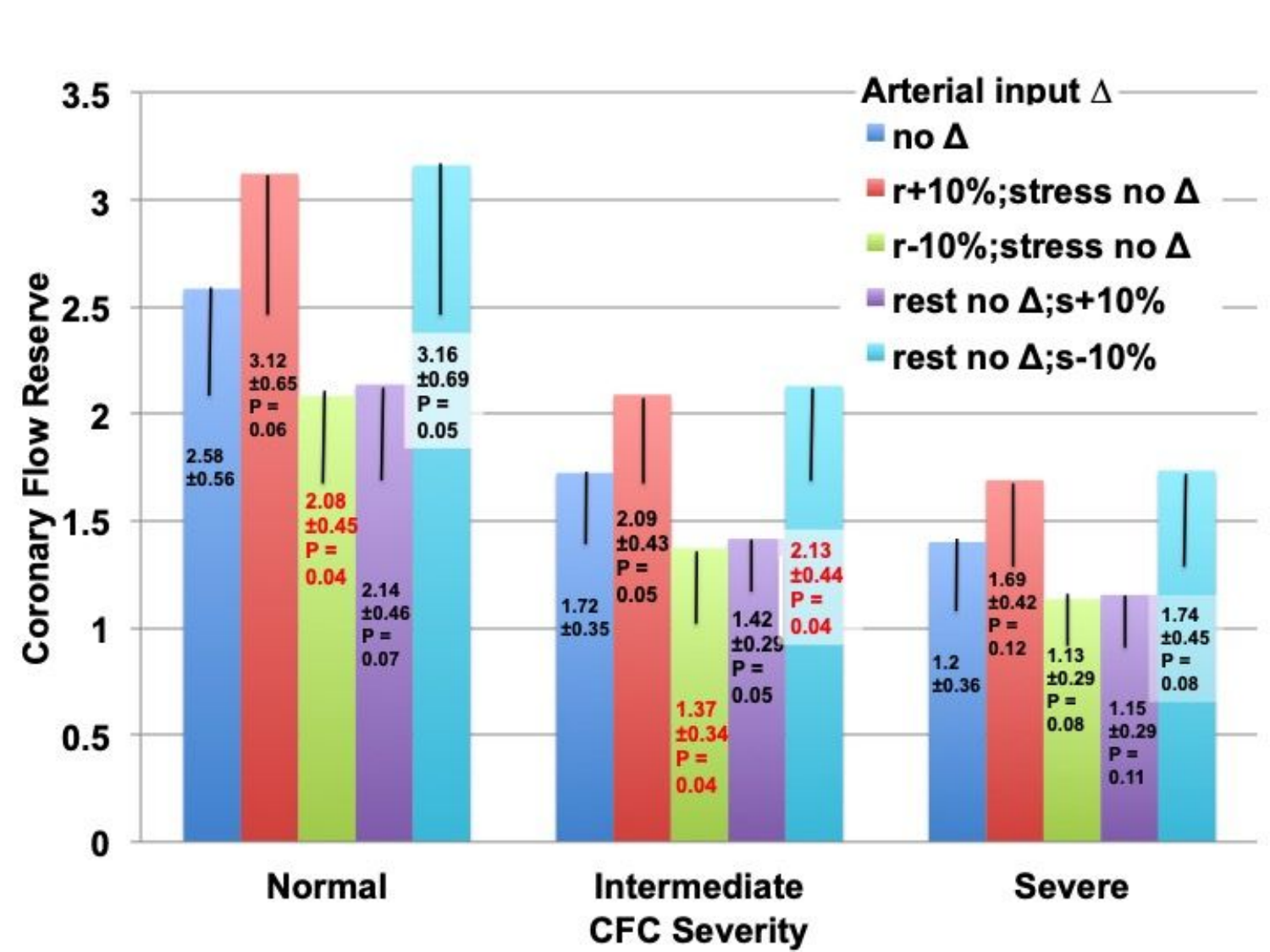

## Slide 5
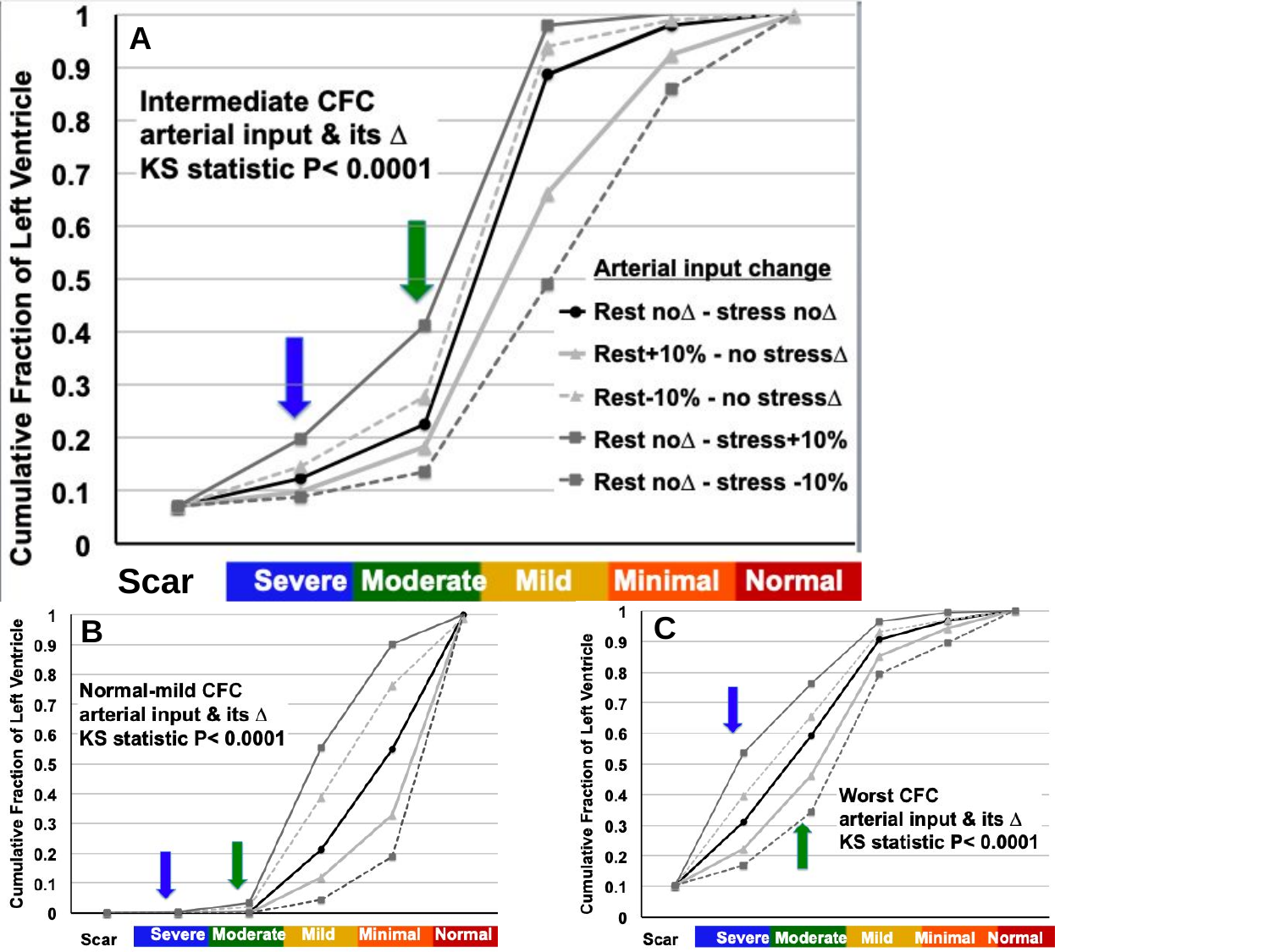

A
Scar
C
B

## Slide 6
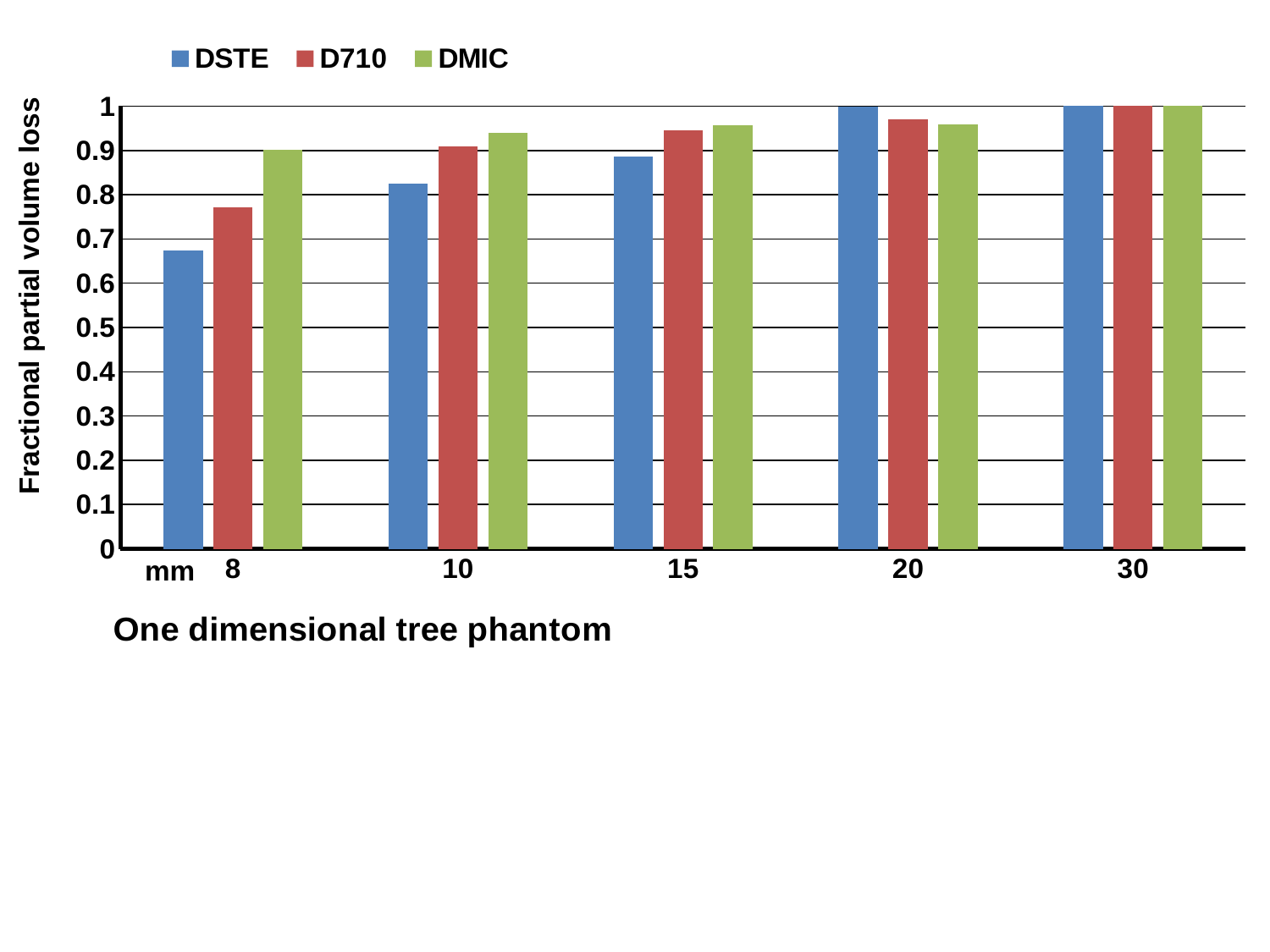

### Chart: One dimensional tree phantom
| Category | DSTE | D710 | DMIC |
|---|---|---|---|
| 8.0 | 0.674756777926507 | 0.771356968349761 | 0.901132852729145 |
| 10.0 | 0.825542180318213 | 0.909638513682969 | 0.938894610367319 |
| 15.0 | 0.88607670750839 | 0.944865629316261 | 0.956402334363199 |
| 20.0 | 0.999559226927479 | 0.970375995683892 | 0.958118777892207 |
| 30.0 | 1.0 | 1.0 | 1.0 |Fractional partial volume loss
mm

## Slide 7
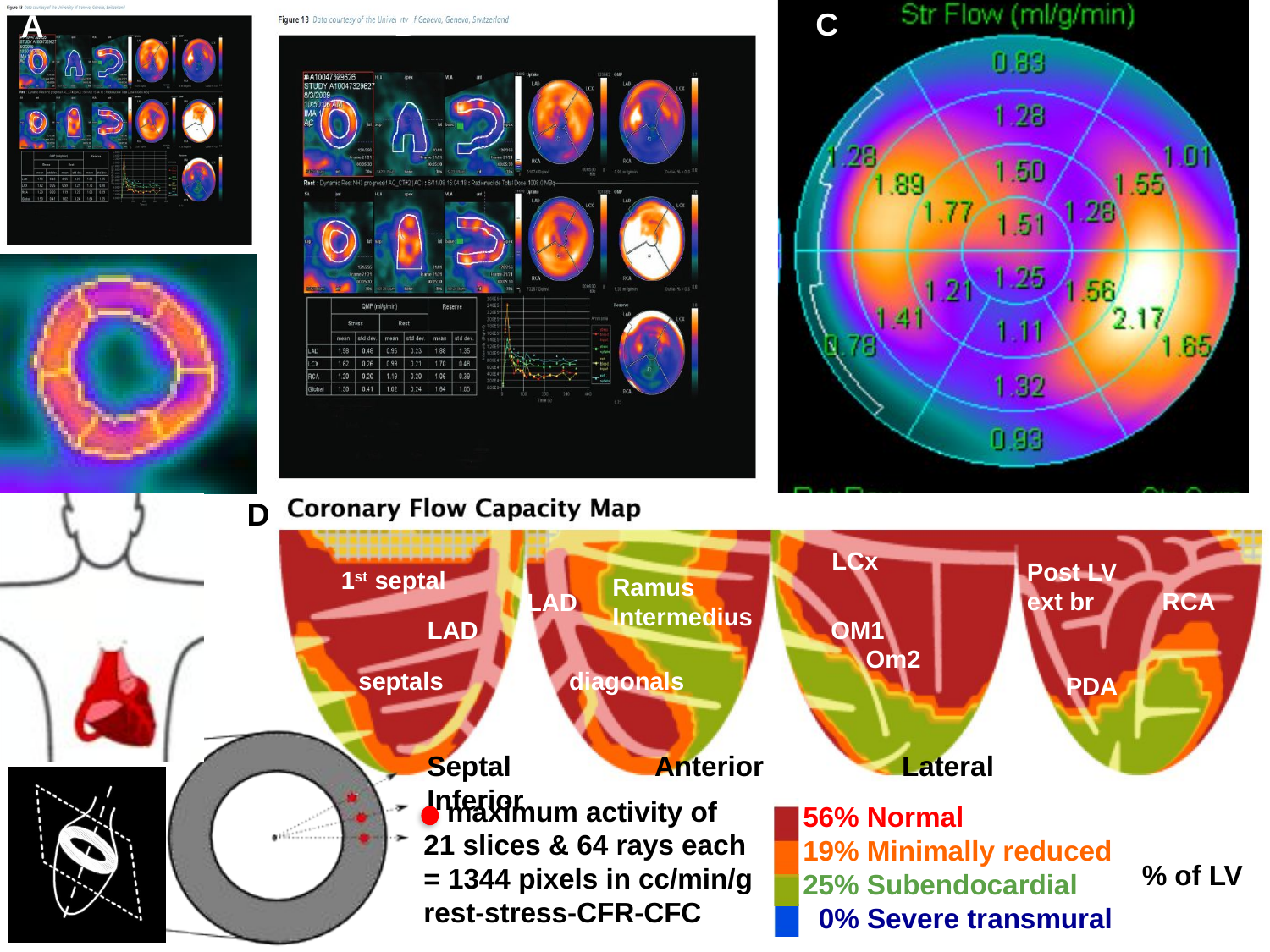

A
B
C
D
LCx
Post LV
ext br
1st septal
Ramus
Intermedius
RCA
LAD
LAD
OM1
 Om2
septals
diagonals
PDA
Septal	 Anterior	 Lateral	 Inferior
 maximum activity of
21 slices & 64 rays each
= 1344 pixels in cc/min/g
rest-stress-CFR-CFC
56% Normal
19% Minimally reduced
25% Subendocardial
 0% Severe transmural
% of LV

## Slide 8
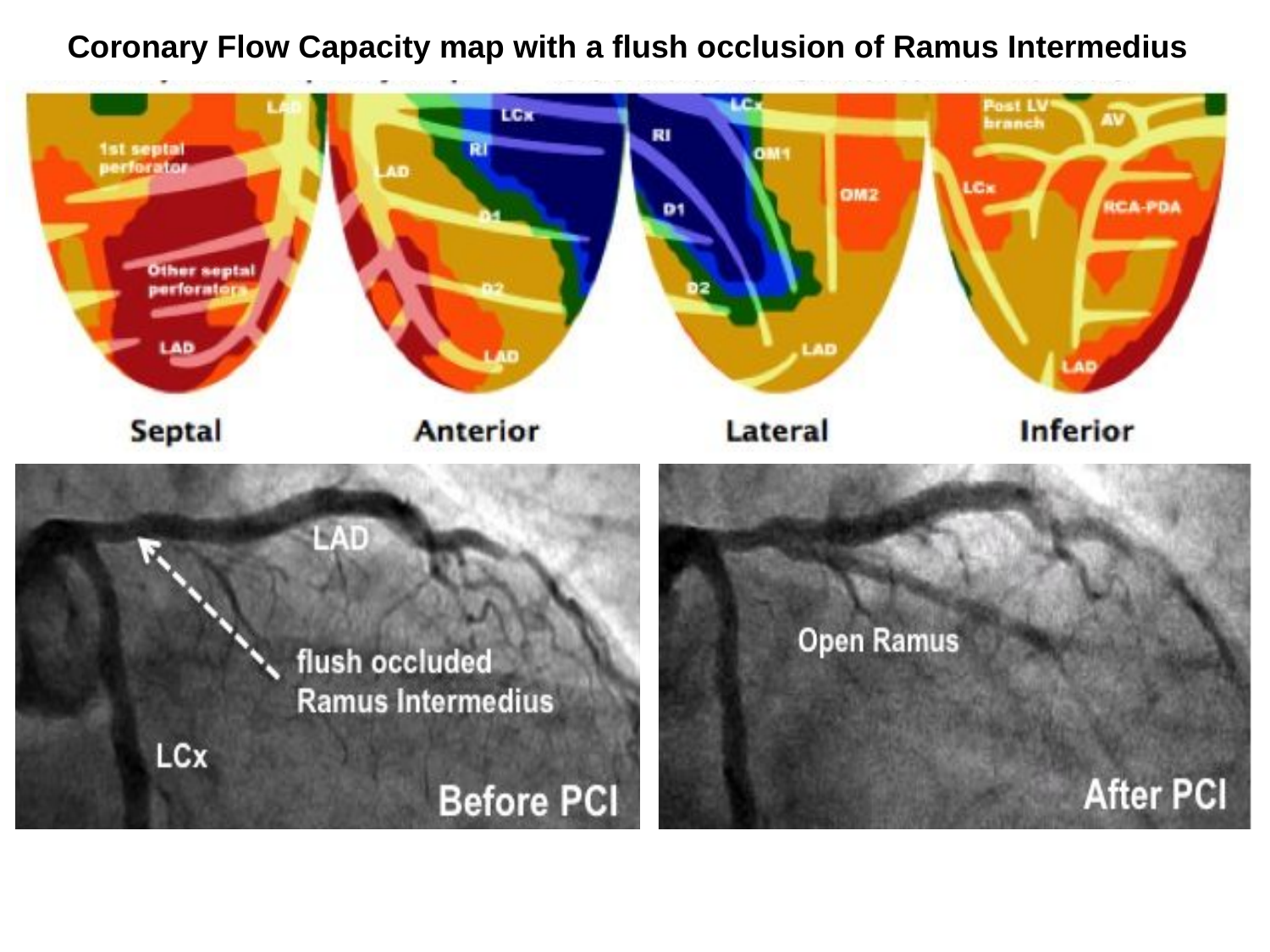

Coronary Flow Capacity map with a flush occlusion of Ramus Intermedius

## Slide 9
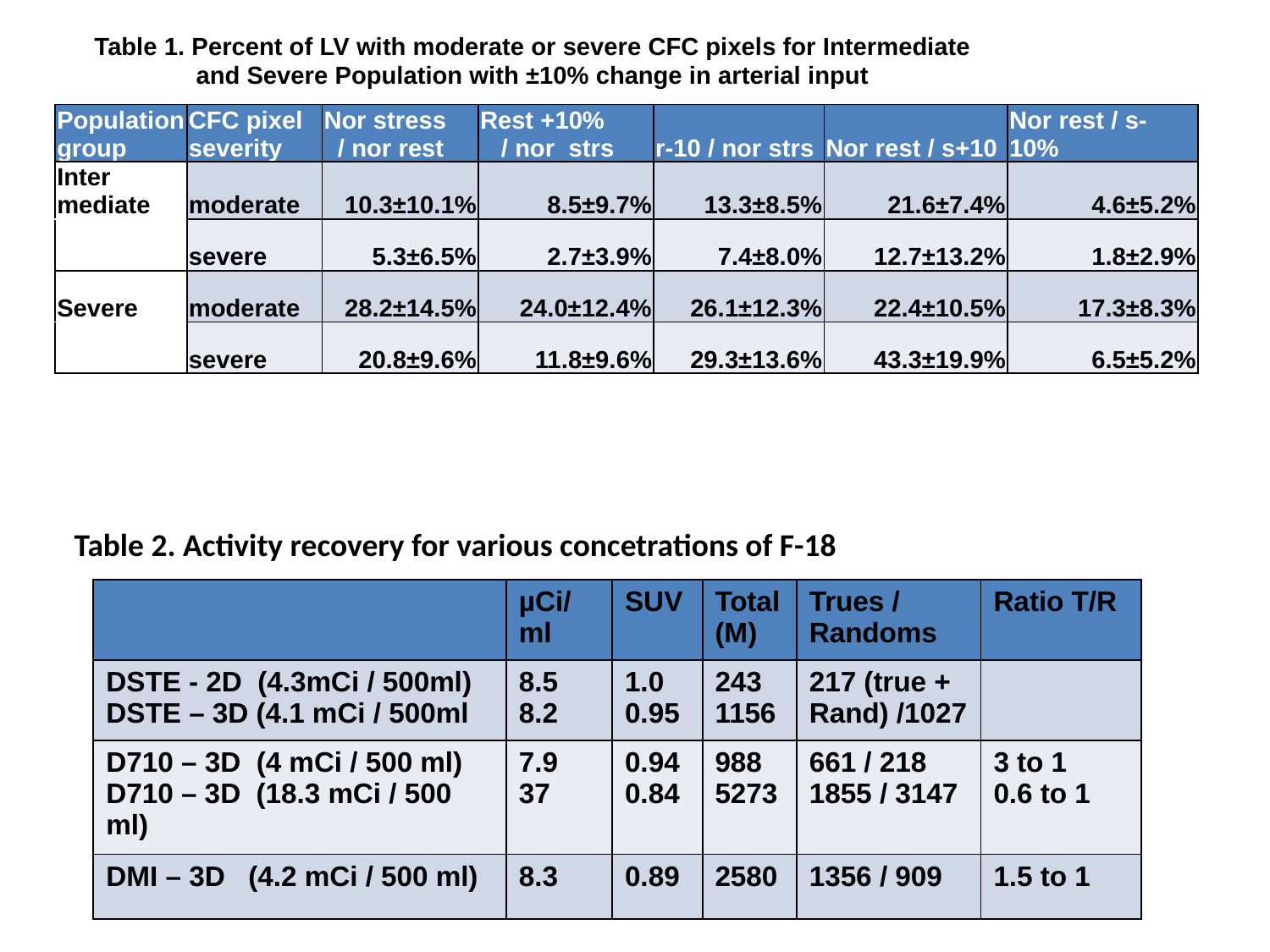

# Table 1. Percent of LV with moderate or severe CFC pixels for Intermediate and Severe Population with ±10% change in arterial input
| Population group | CFC pixel severity | Nor stress / nor rest | Rest +10% / nor strs | r-10 / nor strs | Nor rest / s+10 | Nor rest / s-10% |
| --- | --- | --- | --- | --- | --- | --- |
| Inter mediate | moderate | 10.3±10.1% | 8.5±9.7% | 13.3±8.5% | 21.6±7.4% | 4.6±5.2% |
| | severe | 5.3±6.5% | 2.7±3.9% | 7.4±8.0% | 12.7±13.2% | 1.8±2.9% |
| Severe | moderate | 28.2±14.5% | 24.0±12.4% | 26.1±12.3% | 22.4±10.5% | 17.3±8.3% |
| | severe | 20.8±9.6% | 11.8±9.6% | 29.3±13.6% | 43.3±19.9% | 6.5±5.2% |
Table 2. Activity recovery for various concetrations of F-18
| | µCi/ml | SUV | Total (M) | Trues / Randoms | Ratio T/R |
| --- | --- | --- | --- | --- | --- |
| DSTE - 2D (4.3mCi / 500ml) DSTE – 3D (4.1 mCi / 500ml | 8.5 8.2 | 1.0 0.95 | 243 1156 | 217 (true + Rand) /1027 | |
| D710 – 3D (4 mCi / 500 ml) D710 – 3D (18.3 mCi / 500 ml) | 7.9 37 | 0.94 0.84 | 988 5273 | 661 / 218 1855 / 3147 | 3 to 1 0.6 to 1 |
| DMI – 3D (4.2 mCi / 500 ml) | 8.3 | 0.89 | 2580 | 1356 / 909 | 1.5 to 1 |
Table 2

## Slide 10
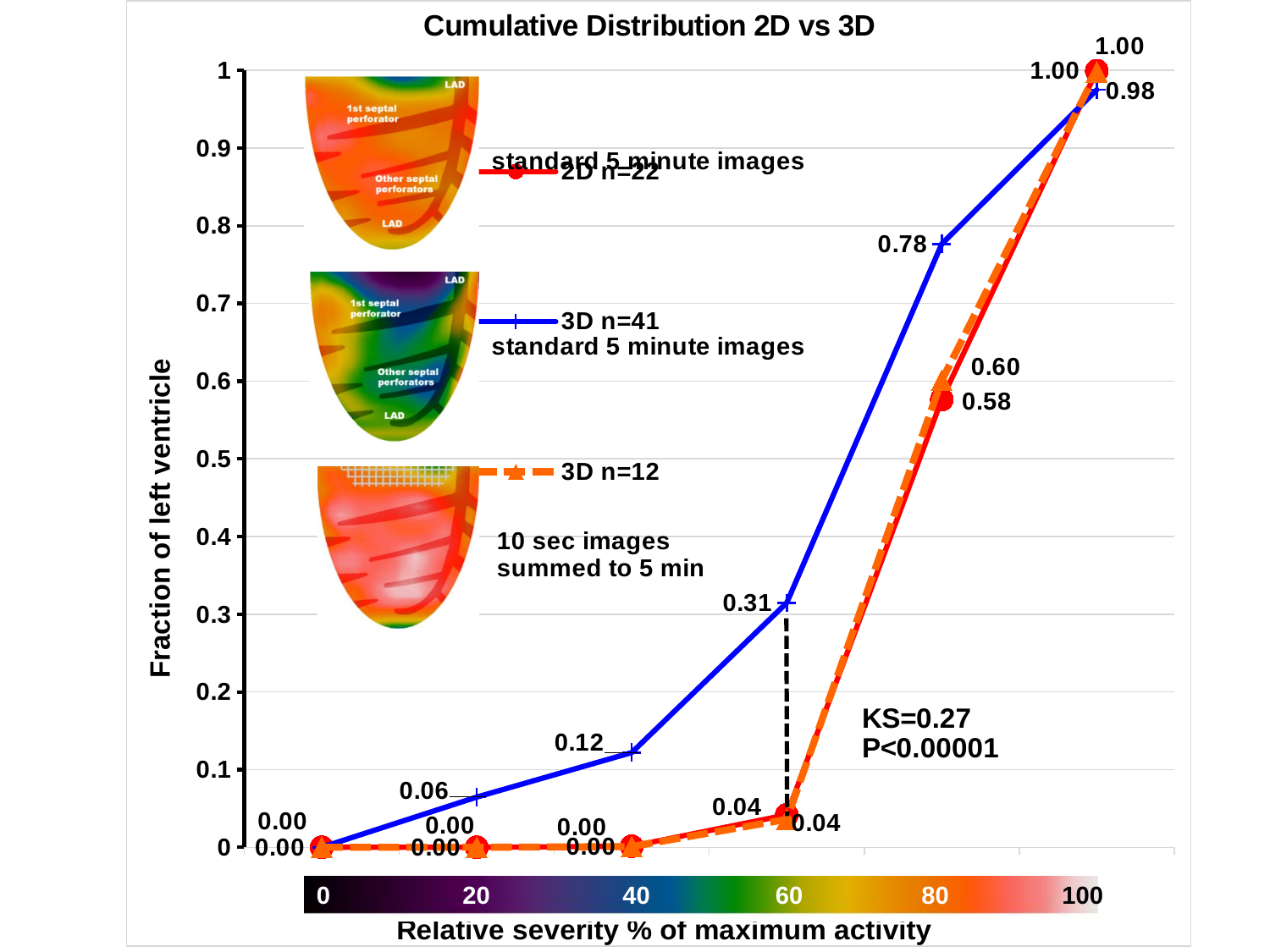

### Chart: Cumulative Distribution 2D vs 3D
| Category | 2D n=22 | 3D n=41 | 3D n=12 |
|---|---|---|---|
| 0 | 0.0 | 0.0 | 0.0 |
| 0-20 | 0.0 | 0.0646341463414634 | 0.0 |
| 20-40 | 0.00136363636363636 | 0.121951219512195 | 0.000902777777777778 |
| 40-60 | 0.0418181818181818 | 0.314634146341463 | 0.0359027777777778 |
| 60-80 | 0.576363636363636 | 0.776585365853658 | 0.600902777777778 |
| 80-100 | 0.999545454545455 | 0.975121951219512 | 0.998402777777778 |
0 20 40 60 80 100

## Slide 11
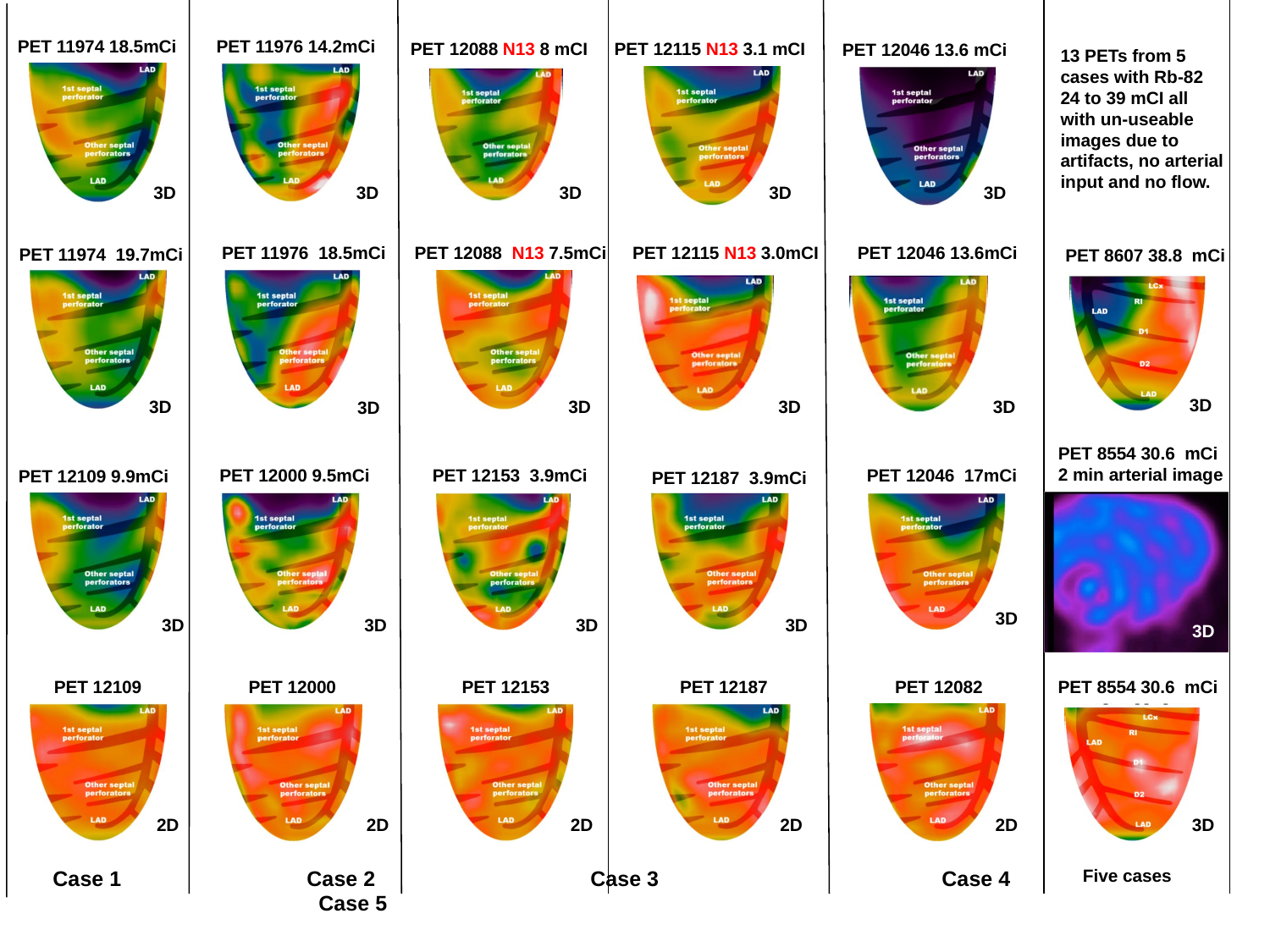

PET 11974 18.5mCi
PET 11976 14.2mCi
PET 12088 N13 8 mCI
PET 12115 N13 3.1 mCI
PET 12046 13.6 mCi
13 PETs from 5
cases with Rb-82
24 to 39 mCI all
with un-useable
images due to
artifacts, no arterial
input and no flow.
3D
3D
3D
3D
3D
PET 11976 18.5mCi
PET 12088 N13 7.5mCi
PET 12115 N13 3.0mCI
PET 12046 13.6mCi
PET 11974 19.7mCi
PET 8607 38.8 mCi
3D
3D
3D
3D
3D
3D
PET 8554 30.6 mCi
2 min arterial image
PET 12000 9.5mCi
PET 12153 3.9mCi
PET 12046 17mCi
PET 12109 9.9mCi
PET 12187 3.9mCi
3D
3D
3D
3D
3D
3D
PET 12109
PET 12187
PET 12082
PET 12000
PET 12153
PET 8554 30.6 mCi
2D
2D
2D
2D
3D
2D
Case 1		Case 2		 Case 3			Case 4		 Case 5
Five cases

## Slide 12
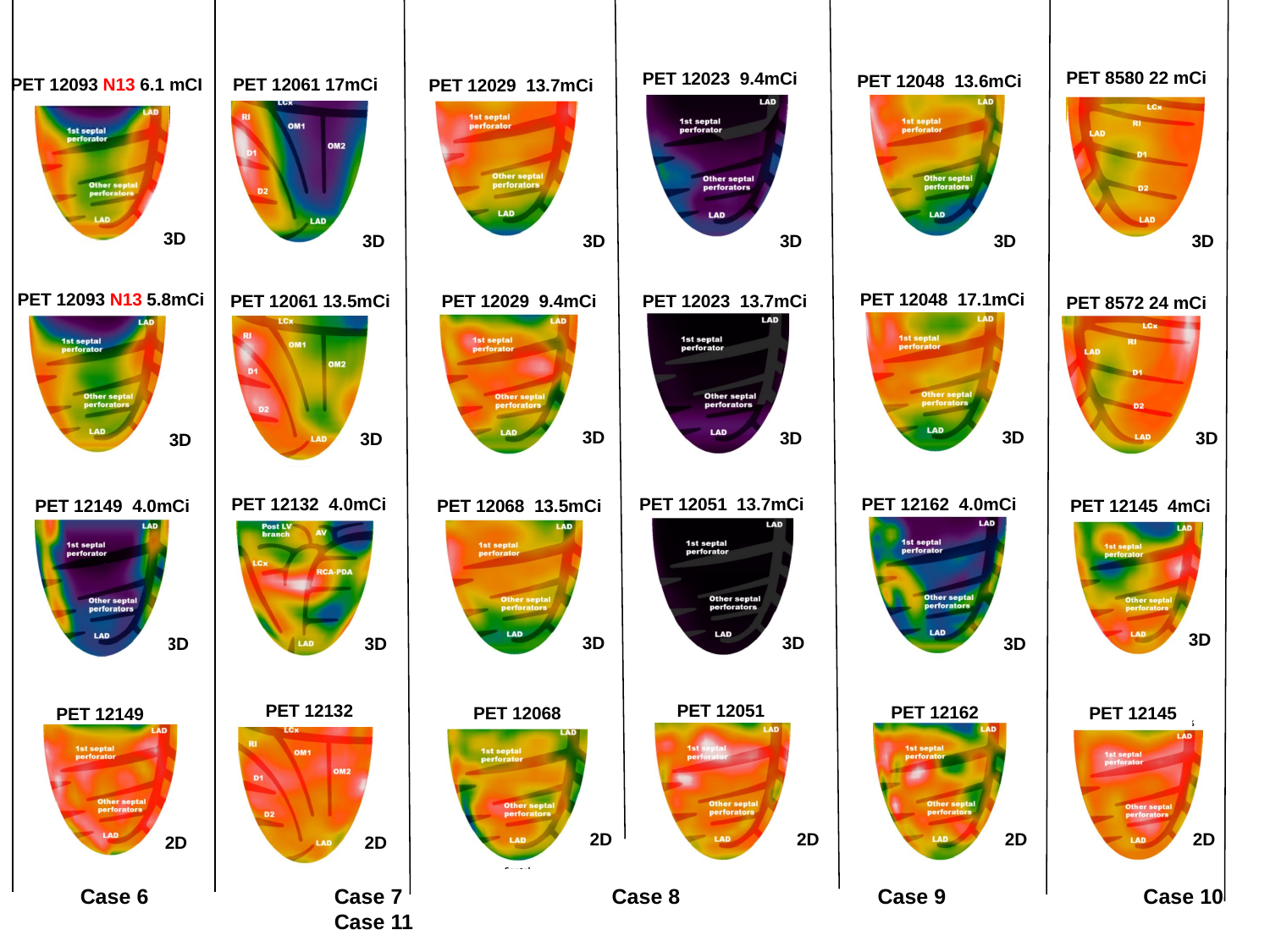

PET 8580 22 mCi
PET 12023 9.4mCi
PET 12048 13.6mCi
PET 12093 N13 6.1 mCI
PET 12061 17mCi
PET 12029 13.7mCi
3D
3D
3D
3D
3D
3D
PET 12093 N13 5.8mCi
PET 12048 17.1mCi
PET 12029 9.4mCi
PET 12023 13.7mCi
PET 12061 13.5mCi
PET 8572 24 mCi
3D
3D
3D
3D
3D
3D
PET 12162 4.0mCi
PET 12132 4.0mCi
PET 12051 13.7mCi
PET 12068 13.5mCi
PET 12145 4mCi
PET 12149 4.0mCi
3D
3D
3D
33D
3D
3D
 PET 12132
PET 12051
PET 12162
 PET 12145
 PET 12068
PET 12149
2D
2D
2D
2D
2D
2D
Case 6		Case 7		 Case 8		 Case 9		 Case 10		Case 11

## Slide 13
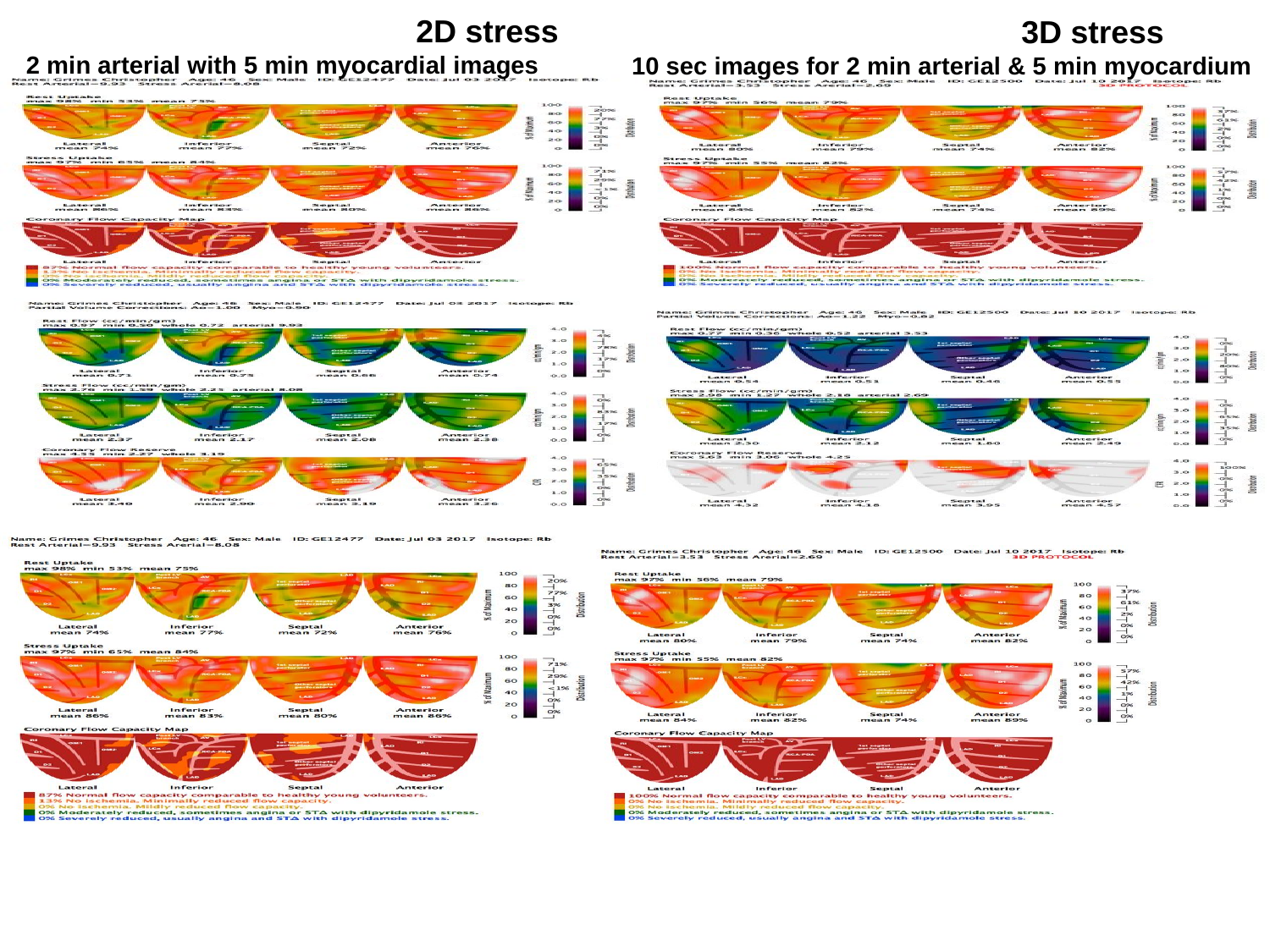

2D stress
2 min arterial with 5 min myocardial images
			 3D stress
10 sec images for 2 min arterial & 5 min myocardium
