# Supplementary material for: Pitfalls in quantitative myocardial PET perfusion II: Arterial input function
Source: J Nucl Cardiol. 2020 Mar 3;27(2):397–409. doi: 10.1007/s12350-020-02074-8 (PMC7174279; doi:10.1007/s12350-020-02074-8)
Supplement: Supplementary file 3 — Electronic supplementary material 3 (PPTX 259 kb) [file 12350_2020_2074_MOESM3_ESM.pptx]

## Slide 1
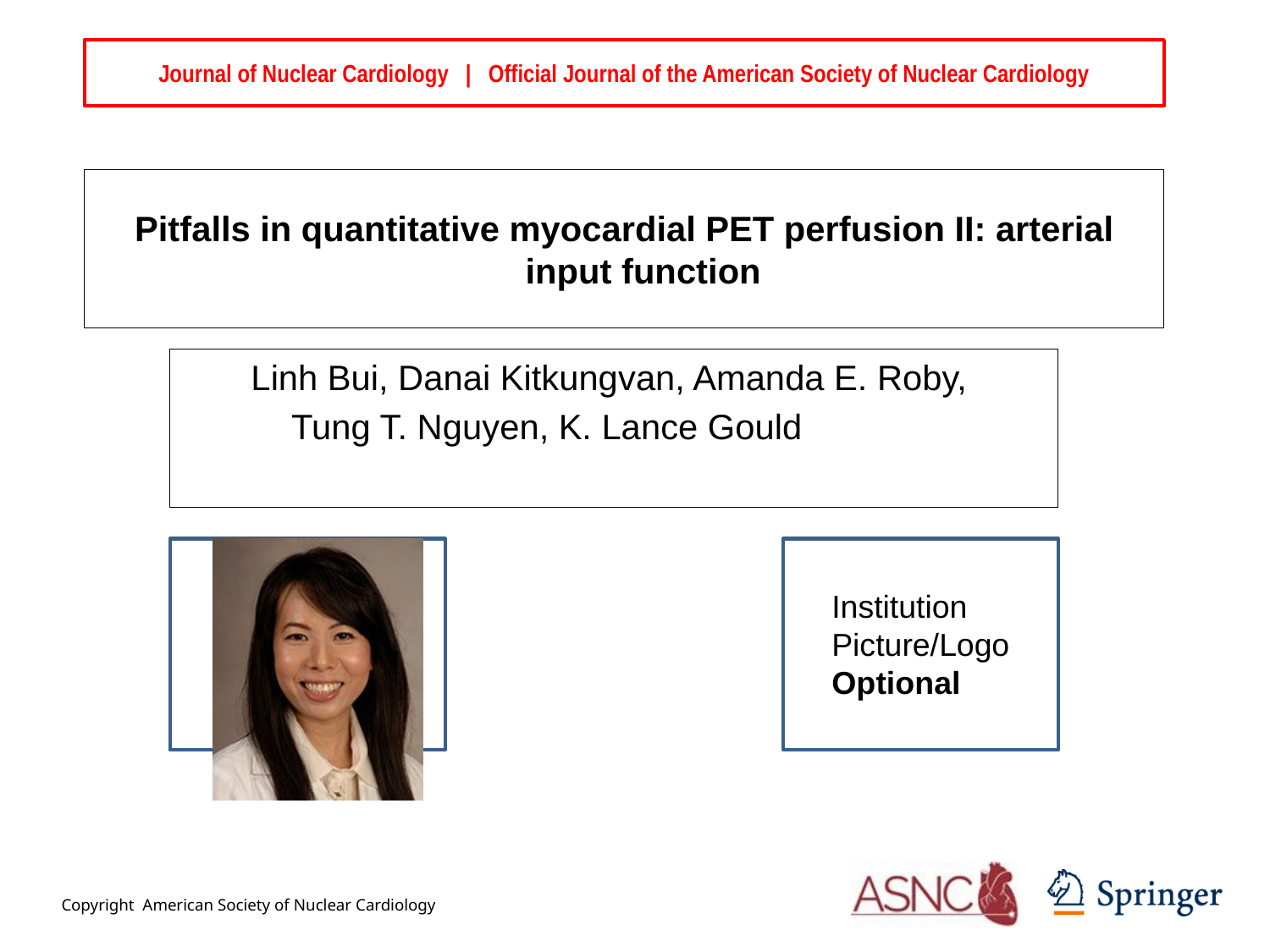

Journal of Nuclear Cardiology | Official Journal of the American Society of Nuclear Cardiology
# Pitfalls in quantitative myocardial PET perfusion II: arterial input function
Linh Bui, Danai Kitkungvan, Amanda E. Roby,
Tung T. Nguyen, K. Lance Gould
Head shot of author
required
Institution
Picture/Logo
Optional
Copyright American Society of Nuclear Cardiology

## Slide 2
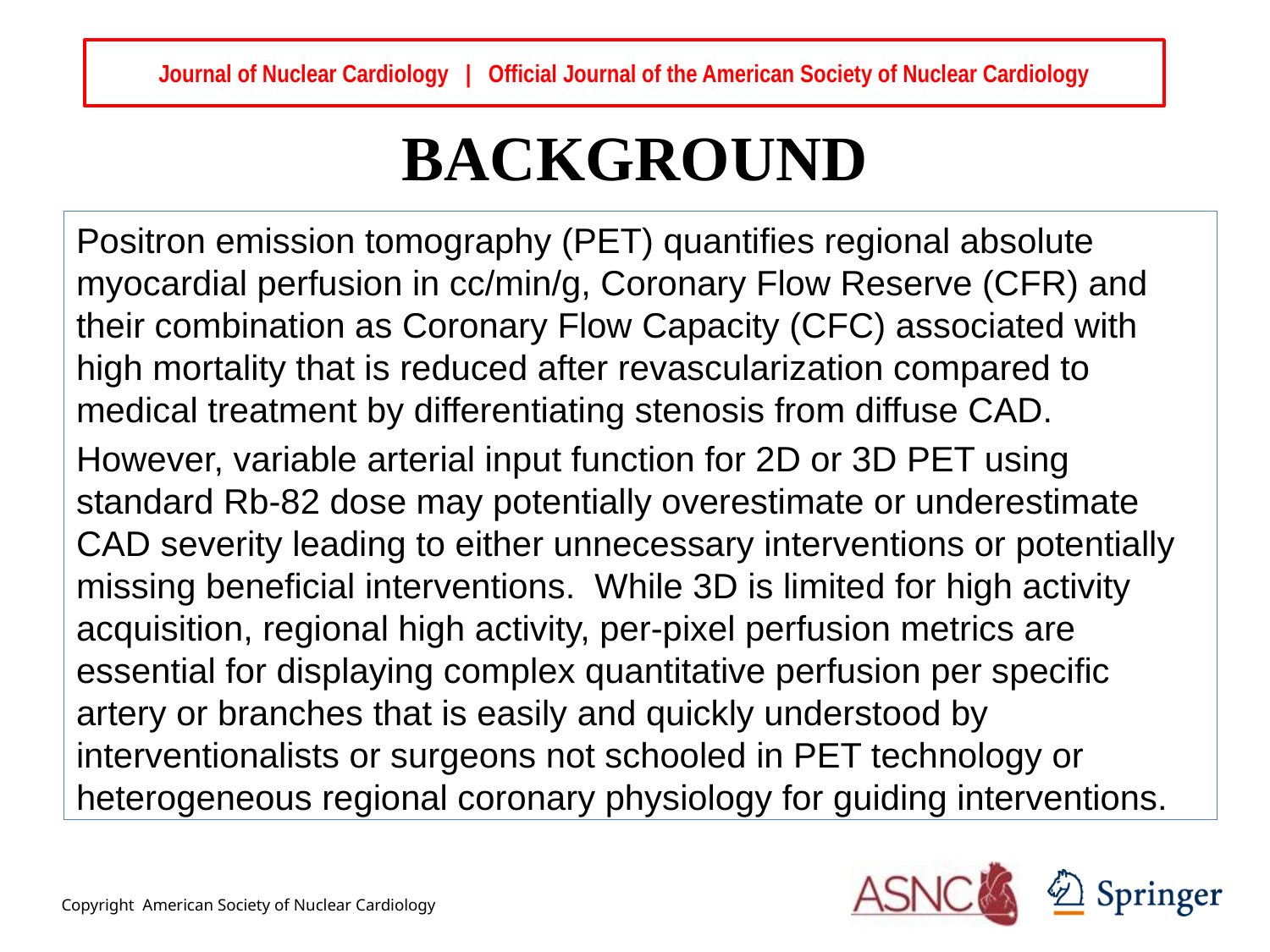

Journal of Nuclear Cardiology | Official Journal of the American Society of Nuclear Cardiology
# BACKGROUND
Positron emission tomography (PET) quantifies regional absolute myocardial perfusion in cc/min/g, Coronary Flow Reserve (CFR) and their combination as Coronary Flow Capacity (CFC) associated with high mortality that is reduced after revascularization compared to medical treatment by differentiating stenosis from diffuse CAD.
However, variable arterial input function for 2D or 3D PET using standard Rb-82 dose may potentially overestimate or underestimate CAD severity leading to either unnecessary interventions or potentially missing beneficial interventions. While 3D is limited for high activity acquisition, regional high activity, per-pixel perfusion metrics are essential for displaying complex quantitative perfusion per specific artery or branches that is easily and quickly understood by interventionalists or surgeons not schooled in PET technology or heterogeneous regional coronary physiology for guiding interventions.
Copyright American Society of Nuclear Cardiology

## Slide 3
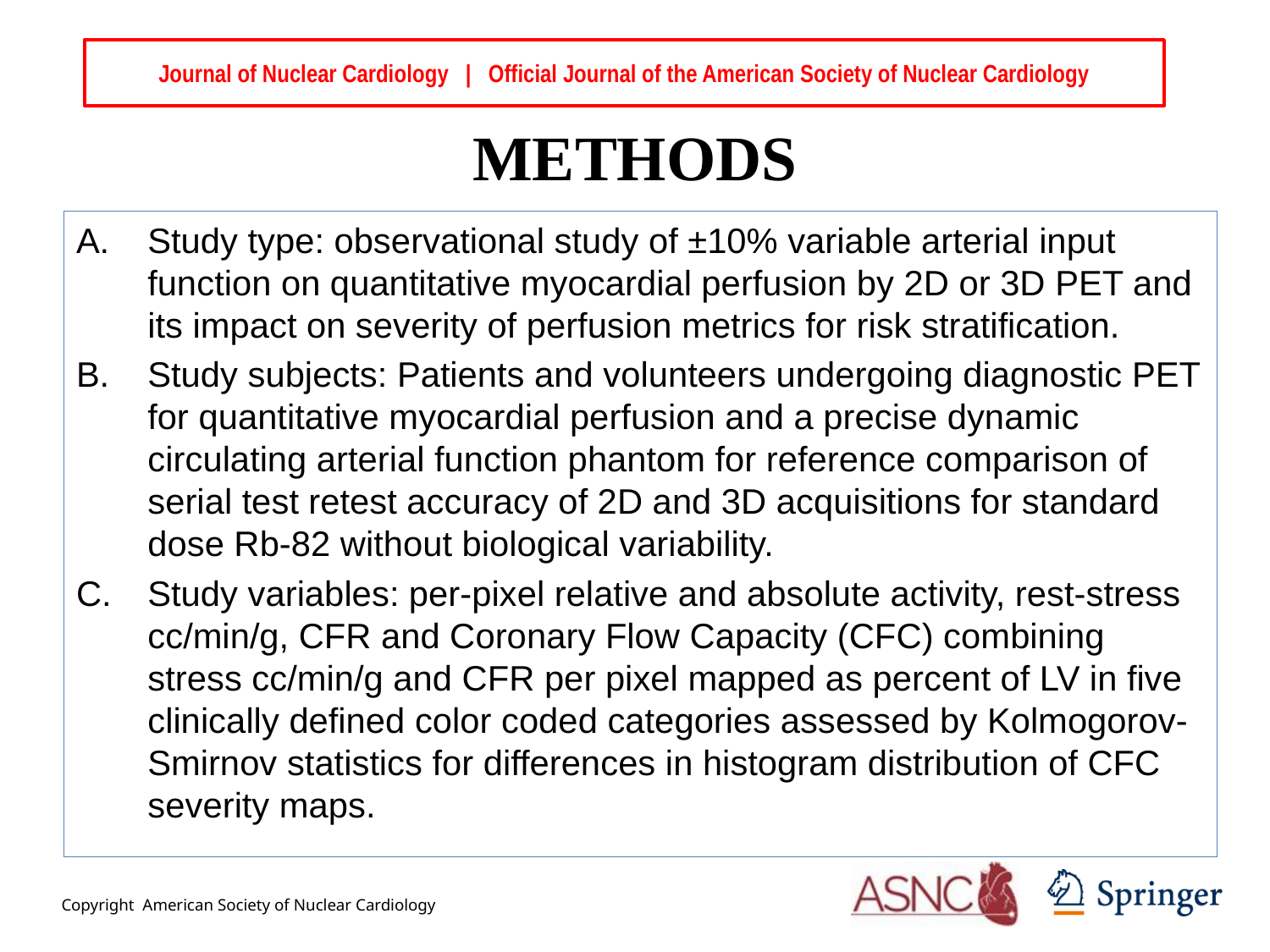

Journal of Nuclear Cardiology | Official Journal of the American Society of Nuclear Cardiology
# METHODS
Study type: observational study of ±10% variable arterial input function on quantitative myocardial perfusion by 2D or 3D PET and its impact on severity of perfusion metrics for risk stratification.
Study subjects: Patients and volunteers undergoing diagnostic PET for quantitative myocardial perfusion and a precise dynamic circulating arterial function phantom for reference comparison of serial test retest accuracy of 2D and 3D acquisitions for standard dose Rb-82 without biological variability.
Study variables: per-pixel relative and absolute activity, rest-stress cc/min/g, CFR and Coronary Flow Capacity (CFC) combining stress cc/min/g and CFR per pixel mapped as percent of LV in five clinically defined color coded categories assessed by Kolmogorov-Smirnov statistics for differences in histogram distribution of CFC severity maps.
Copyright American Society of Nuclear Cardiology

## Slide 4
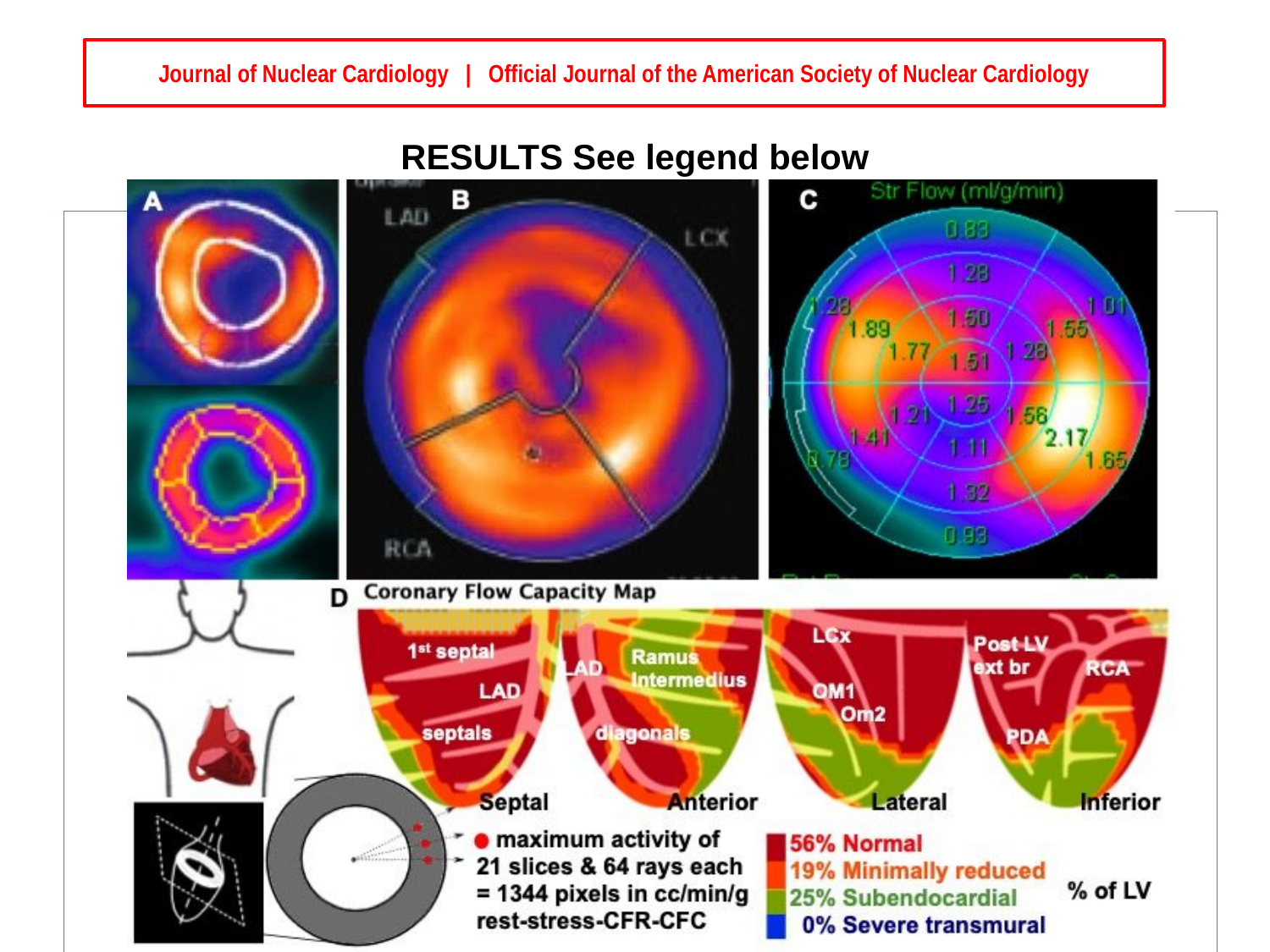

Journal of Nuclear Cardiology | Official Journal of the American Society of Nuclear Cardiology
# RESULTS See legend below

## Slide 5
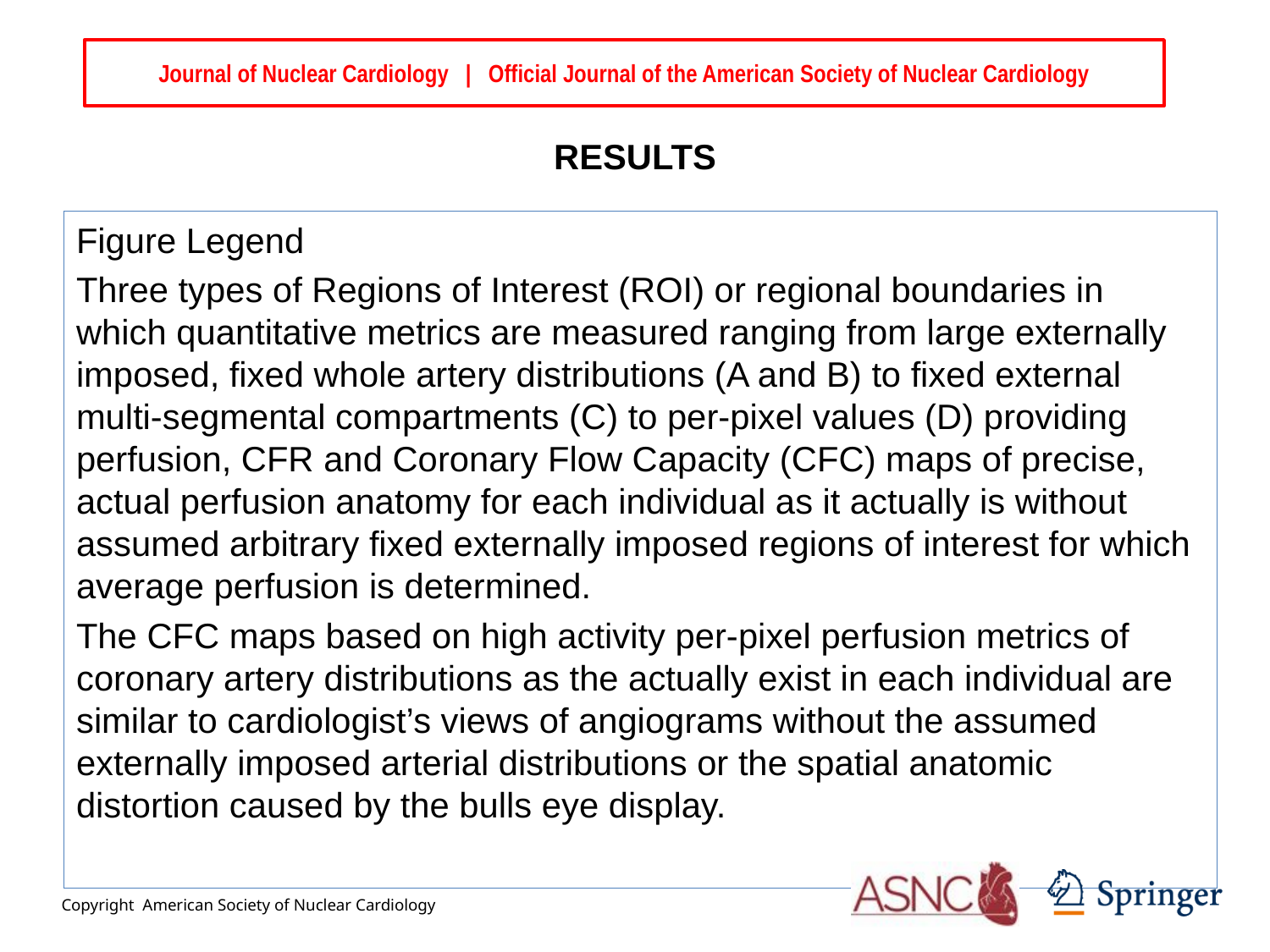

Journal of Nuclear Cardiology | Official Journal of the American Society of Nuclear Cardiology
# RESULTS
Figure Legend
Three types of Regions of Interest (ROI) or regional boundaries in which quantitative metrics are measured ranging from large externally imposed, fixed whole artery distributions (A and B) to fixed external multi-segmental compartments (C) to per-pixel values (D) providing perfusion, CFR and Coronary Flow Capacity (CFC) maps of precise, actual perfusion anatomy for each individual as it actually is without assumed arbitrary fixed externally imposed regions of interest for which average perfusion is determined.
The CFC maps based on high activity per-pixel perfusion metrics of coronary artery distributions as the actually exist in each individual are similar to cardiologist’s views of angiograms without the assumed externally imposed arterial distributions or the spatial anatomic distortion caused by the bulls eye display.
Copyright American Society of Nuclear Cardiology

## Slide 6
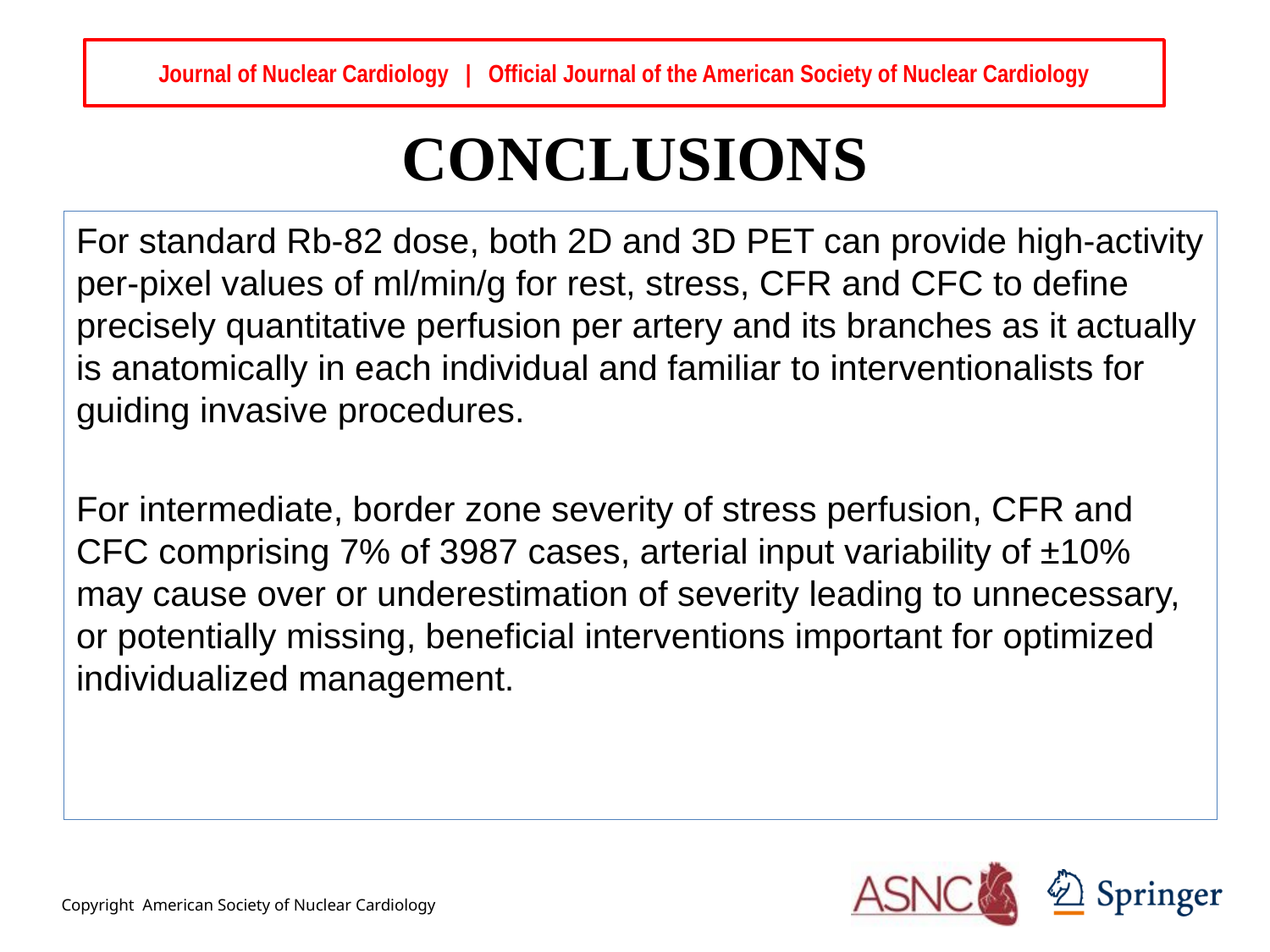

Journal of Nuclear Cardiology | Official Journal of the American Society of Nuclear Cardiology
# CONCLUSIONS
For standard Rb-82 dose, both 2D and 3D PET can provide high-activity per-pixel values of ml/min/g for rest, stress, CFR and CFC to define precisely quantitative perfusion per artery and its branches as it actually is anatomically in each individual and familiar to interventionalists for guiding invasive procedures.
For intermediate, border zone severity of stress perfusion, CFR and CFC comprising 7% of 3987 cases, arterial input variability of ±10% may cause over or underestimation of severity leading to unnecessary, or potentially missing, beneficial interventions important for optimized individualized management.
Copyright American Society of Nuclear Cardiology
